# A chromosome-level genome assembly of the oriental river prawn, *Macrobrachium nipponense*

**DOI:** 10.1093/gigascience/giaa160

**Published:** 2021-01-18

**Authors:** Shubo Jin, Chao Bian, Sufei Jiang, Kai Han, Yiwei Xiong, Wenyi Zhang, Chengcheng Shi, Hui Qiao, Zijian Gao, Ruihan Li, Yu Huang, Yongsheng Gong, Xinxin You, Guangyi Fan, Qiong Shi, Hongtuo Fu

**Affiliations:** Key Laboratory of Freshwater Fisheries and Germplasm Resources Utilization, Ministry of Agriculture, Freshwater Fisheries Research Center, Chinese Academy of Fishery Sciences, Wuxi 214081, China; Shenzhen Key Lab of Marine Genomics, Guangdong Provincial Key Lab of Molecular Breeding in Marine Economic Animals, BGI Academy of Marine Sciences, BGI Marine, BGI, Shenzhen 518083, China; Key Laboratory of Freshwater Fisheries and Germplasm Resources Utilization, Ministry of Agriculture, Freshwater Fisheries Research Center, Chinese Academy of Fishery Sciences, Wuxi 214081, China; BGI-Qingdao, BGI-Shenzhen, Qingdao 266555, China; Key Laboratory of Freshwater Fisheries and Germplasm Resources Utilization, Ministry of Agriculture, Freshwater Fisheries Research Center, Chinese Academy of Fishery Sciences, Wuxi 214081, China; Key Laboratory of Freshwater Fisheries and Germplasm Resources Utilization, Ministry of Agriculture, Freshwater Fisheries Research Center, Chinese Academy of Fishery Sciences, Wuxi 214081, China; BGI-Qingdao, BGI-Shenzhen, Qingdao 266555, China; Key Laboratory of Freshwater Fisheries and Germplasm Resources Utilization, Ministry of Agriculture, Freshwater Fisheries Research Center, Chinese Academy of Fishery Sciences, Wuxi 214081, China; Shenzhen Key Lab of Marine Genomics, Guangdong Provincial Key Lab of Molecular Breeding in Marine Economic Animals, BGI Academy of Marine Sciences, BGI Marine, BGI, Shenzhen 518083, China; Shenzhen Key Lab of Marine Genomics, Guangdong Provincial Key Lab of Molecular Breeding in Marine Economic Animals, BGI Academy of Marine Sciences, BGI Marine, BGI, Shenzhen 518083, China; Shenzhen Key Lab of Marine Genomics, Guangdong Provincial Key Lab of Molecular Breeding in Marine Economic Animals, BGI Academy of Marine Sciences, BGI Marine, BGI, Shenzhen 518083, China; Key Laboratory of Freshwater Fisheries and Germplasm Resources Utilization, Ministry of Agriculture, Freshwater Fisheries Research Center, Chinese Academy of Fishery Sciences, Wuxi 214081, China; Shenzhen Key Lab of Marine Genomics, Guangdong Provincial Key Lab of Molecular Breeding in Marine Economic Animals, BGI Academy of Marine Sciences, BGI Marine, BGI, Shenzhen 518083, China; BGI-Qingdao, BGI-Shenzhen, Qingdao 266555, China; Shenzhen Key Lab of Marine Genomics, Guangdong Provincial Key Lab of Molecular Breeding in Marine Economic Animals, BGI Academy of Marine Sciences, BGI Marine, BGI, Shenzhen 518083, China; Key Laboratory of Freshwater Fisheries and Germplasm Resources Utilization, Ministry of Agriculture, Freshwater Fisheries Research Center, Chinese Academy of Fishery Sciences, Wuxi 214081, China

**Keywords:** Macrobrachium nipponense, Chromosome-level genome, Evolutionary analysis, Genome duplication, Candidate sex-related genes

## Abstract

**Background:**

The oriental river prawn, *Macrobrachium nipponense*, is an economically important shrimp in China. Male prawns have higher commercial value than females because the former grow faster and reach larger sizes. It is therefore important to reveal sex-differentiation and development mechanisms of the oriental river prawn to enable genetic improvement.

**Results:**

We sequenced 293.3 Gb of raw Illumina short reads and 405.7 Gb of Pacific Biosciences long reads. The final whole-genome assembly of the Oriental river prawn was ∼4.5 Gb in size, with predictions of 44,086 protein-coding genes. A total of 49 chromosomes were determined, with an anchor ratio of 94.7% and a scaffold N50 of 86.8 Mb. A whole-genome duplication event was deduced to have happened 109.8 million years ago. By integration of genome and transcriptome data, 21 genes were predicted as sex-related candidate genes.

**Conclusion:**

The first high-quality chromosome-level genome assembly of the oriental river prawn was obtained. These genomic data, along with transcriptome sequences, are essential for understanding sex-differentiation and development mechanisms in the oriental river prawn, as well as providing genetic resources for in-depth studies on developmental and evolutionary biology in arthropods.

## Introduction

**Figure 1:**
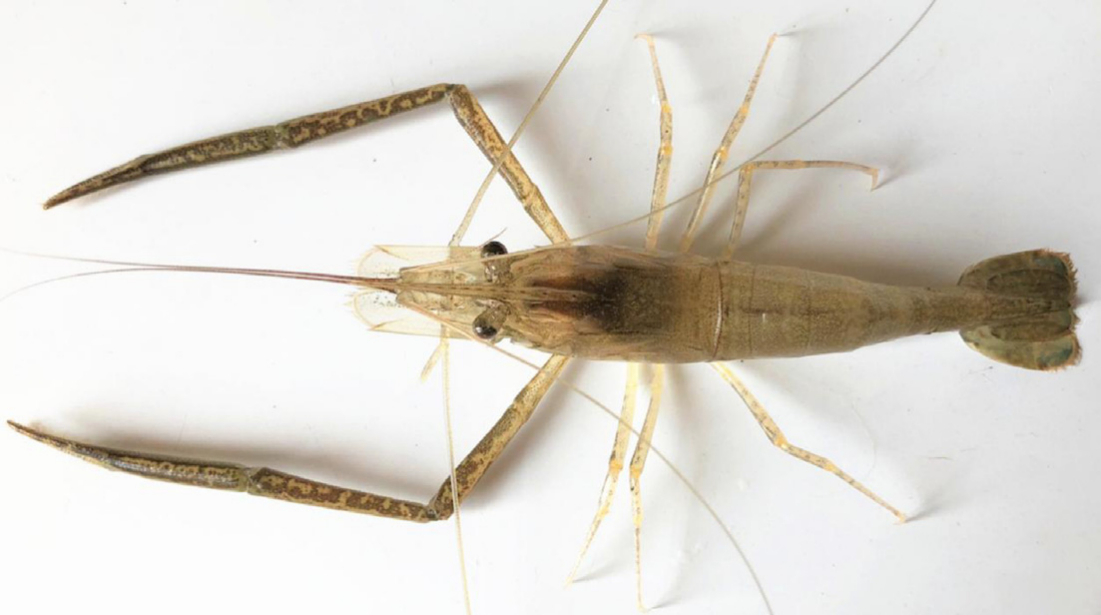
Picture of the sequenced oriental river prawn. This individual was collected from Taihu Lake, Wuxi City, Jiangsu Province, China.

The oriental river prawn, *Macrobrachium nipponense* (NCBI:txid159736; marinespecies.org: taxname:587405; Subphylum Crustacea, Order Decapoda, Family Palaemonidae; Fig. 1), is widely distributed in freshwater and low-salinity estuarine regions of China [1, 2]. It has become an important commercial species in China owing to its high nutritional value and delicious taste. Its annual production has gradually increased in recent years (up to 272,592 tons in 2016) [3]. The annual output value was ∼2.8 billion US dollars.

It is notable that the growth performance of the oriental river prawn shows remarkable differences between male and female individuals. Males usually grow faster than their female counterparts and reach larger sizes at the harvest period each year. Thus, culturing all-male populations is a long-term goal for practical aquaculture and will lead to a dramatic increase in economic profits. In addition, our previous study [3] reported that both ovaries and testes in oriental river prawns begin to differentiate at a post-developmental stage (day 13; PL13), and mature at PL19 to PL22, on the basis of histological observations and steroid hormone levels. In practice, quick gonad development restricts sustainable growth of the oriental river prawn industry because over-reproduction will happen frequently during the farming process, leading to poor survival, low growth rates, and small body size. Therefore, it is critical to understand the mechanism of sexual differentiation and reproductive development in the oriental river prawn to obtain genetic improvement.

In the present study, a chromosome-level genome assembly for the oriental river prawn was constructed by integration of Pacific Biosciences (PacBio) long reads, Illumina short reads, and Hi-C sequencing data. These genomic data along with transcriptome sequences will be a useful resource for in-depth studies on sex differentiation and the mechanism of reproduction in the oriental river prawn, as well as promoting comparative genomic analyses with other prawn species.

## Methods

### Sample collection

Specimens of oriental river prawn were collected from a wild population in Tai Lake, Wuxi, China (120 13.44 E, 31 28.22 N). In a lineage family, 1 individual with a body weight of 14.01 g was selected for a *k*-mer analysis, 5 individuals with body weights of 13.02–15.56 g were selected for Illumina sequencing, and another 5 individuals with body weights of 10.50–13.06 g were selected for PacBio sequencing. Fresh muscle tissues of multiple individuals from both groups were collected and immediately frozen in liquid nitrogen before carrying out DNA extraction.

### DNA extraction and whole-genome sequencing

Muscle tissues from the 5 individuals in each group were pooled, and then genomic DNAs (gDNAs) from the pooled samples were extracted using a Nucleic Acid Kit (Qiagen, Germantown, MD, USA) in accordance with the manufacturer's instructions. gDNAs were also extracted from the muscle of a single individual for the *k*-mer analysis. The extracted gDNA was then used to construct libraries for Illumina (Illumina Inc., San Diego, CA, USA) and PacBio (Menlo Park, CA, USA) sequencing. According to the Illumina protocols, 2 short paired-end libraries (with insert sizes of 500 and 800 bp, respectively) were constructed for the *k*-mer analysis. Another 7 paired-end libraries (with insert sizes of 270, 500, and 800 bp and 2, 5, 10, and 20 kb, respectively) were constructed for the whole-genome sequencing. These libraries were then sequenced on an Illumina HiSeq X-Ten platform (PE150 in length) (Illumina HiSeq X Ten, RRID:SCR_016385). Approximately 157.3 Gb of raw reads were generated for the *k*-mer analysis, and ∼293.3 Gb of raw sequenced reads were produced for the whole-genome assembly. Subsequently, 253.4 Gb of clean data were retained for assembly through filtering low-quality data and removal of duplicated reads and adapter sequences by SOAPfilter v2.2 (SOAPfilter, RRID:SCR_014986) [4]. In parallel to this, long inserted libraries were created using the PacBio Sequel platform (PacBio Sequel System, RRID:SCR_017989) for the whole-genome assembly. Approximately 405.7 Gb of PacBio long reads were generated. These long reads were then corrected by LoRDEC (LoRDEC, RRID:SCR_015814) [5] using default parameters.

### RNA extraction and transcriptome sequencing

Male individuals of oriental river prawn in both reproductive and non-reproductive seasons were also collected from a wild population in Tai Lake, Wuxi, China. Shrimp in the non-reproductive season with body weights of 2.54–5.08 g were collected in January 2018 (water temperature ≤15°C, light cycle ≤10 h), while shrimps in the reproductive season with body weights of 3.07–5.24 g were collected in July 2018 (water temperature of ≥28°C, light cycle ≥16 h).

All the prawns were transferred to a 500-L indoor tank with aerated fresh water for 2 days before tissue collection. Testes and androgenic glands were collected from specimens in the non-reproductive season and reproductive season. At least 0.5 g of testes and androgenic glands (n > 50) were pooled to form 1 biological replicate, and 3 biological replicates were separated for transcriptome sequencing. These collected samples were immediately frozen in liquid nitrogen and stored at −80°C until use.

Total RNA was extracted using a UNlQ-10 Column Trizol Total RNA Isolation Kit (Sangon Biotech, Shanghai, China) following the manufacturer's protocol. In brief, the total messenger RNAs were digested individually into fragments, and 200-bp raw paired-end reads were generated using the Illumina sequencing platform.

### Hi-C library preparation

To construct pseudo-chromosomes, another 10 male individuals of oriental river prawn with body weights of 10.16–13.4 g were collected from the same lineage. Blood samples of these individuals were also collected. A Hi-C library was constructed with the pool of extracted blood gDNAs, and it was then sequenced on an Illumina HiSeq X-Ten platform.

### Genome assembly

The genome size of the oriental river prawn was estimated by using a routine 17-mer frequency distribution analysis [6] on cleaned Hiseq data with insert sizes of 500 and 800 bp. The genome size was calculated according to the following equation: genome size = *k*-mer number/expected *k*-mer depth. In the case of sufficient data, the *k*-mer frequency distribution follows a Poisson distribution pattern, and the peak of the *k*-mer distribution curve is considered as the expectation of *k*-mer depth. As a result, the genome size of the oriental river prawn was estimated to be ∼4.6 Gb.

Long reads sequenced using the PacBio platform were assembled by using the Shasta long read assembler v0.2.0 [7] with 200 minimal component size (“–ReadGraph.min ComponentSize”) to ensure that the best-quality read graph and 50 minimal aligned markers (“–Align.min AlignedMarkerCount”) matched aligned read pairs. The consensus caller model was set as “Modal” to assemble repeat counts. Those paired-end reads with an insert size of 200–800 bp were aligned against the draft assembly using BWA v0.7.12 (BWA, RRID:SCR_010910) [8], and the assembled sequences were then improved through 2 rounds of polishing using Pilon v1.23 (Pilon, RRID:SCR_014731) [9] based on the read alignments.

To improve the draft genome assembly to a chromosome level, a Hi-C library was constructed following the method of Rao et al. [10] using pooled blood gDNAs. Chromatins were cross-linked with formaldehyde and digested with MboI enzyme; subsequently, the generated sticky ends were filled and further ligated, and then DNAs were purified and sheared. Paired-end sequencing was performed using the BGI-seq 500 platform (BGI, Shenzhen, China). First, all valid read pairs were extracted on the basis of the results of Hic-Pro v2.8.0 (Hic-Pro, RRID:SCR_017643) [11] and further aligned to the draft genome assembly using Juicer v1.5 (Juicer, RRID:SCR_017226) [12] to generate the Hi-C interaction maps (“merged_nodups.txt” file). Subsequently, scaffold sequences from the draft assembly were ordered and oriented using the 3D-DNA pipeline [13] to be integrated into long pseudo-chromosomes. Manual review and refinement were performed for identification and removal of assembly errors with assistance of the Juicebox Assembly Tool v1.9.0 [14].

### Repeat and gene structure annotation

Two routine approaches, including *ab initio* and homology prediction methods, were used to detect repetitive elements in the genome assembly. In the *ab initio* prediction, RepeatModeler v1.04 (RepeatModeler, RRID:SCR_015027) [15] and LTR-FINDER v1.06 (LTR-FINDER, RRID:SCR_015247) [16] were used with default parameters to detect repetitive elements. Then, a *de novo* repeat sequence library was built using the results. Subsequently, RepeatMasker (RepeatMasker, RRID:SCR_012954) [17] was used to annotate the novel library based on Repbase TE (v14.04) [18]. Additionally, Tandem Repeats Finder (v4.04) [19] was applied to identify the tandem elements. For the homology prediction, RepeatMasker [17] and RepeatProteinMask (v3.2.2) [17] were used to search the repeat elements among the assembled genome based on RepBase TE (v14.04) [18]. After combining the results from the 2 aforementioned approaches, it was found that repeat sequences accounted for ∼50.2% of the assembled genome. Finally, repeat regions were masked in the genome of the oriental river prawn for prediction of protein-coding genes.

An integration of 3 methods, including *de novo* prediction, homology-based annotation, and transcriptome-based annotation, was applied to predict protein-coding genes in the assembled genome. For the *de novo* prediction, Augustus v3.0.2 (Augustus, RRID:SCR_008417) [20] was performed to identify coding regions on the repeat-masked assembly with default parameters. For the homology-based prediction, protein sequences of 9 representative species (*Caenorhabditis elegans, Eriocheir sinensis, Danio rerio, Daphnia pulex, Drosophila melanogaster, Homo sapiens, Crassostrea gigas, Pinctada fucata martensii*, and *Litopenaeus vannamei*) downloaded from the NCBI database were mapped onto the oriental river prawn genome using TBLASTn v2.2.25 (TBLASTn, RRID:SCR_001010) [21] with an e-value ≤ 10^−5^. Subsequently, GeneWise v2.2.0 (GeneWise, RRID:SCR_015054) [22] was applied to identify gene structures based on the best TBLASTn alignments. For the transcriptome-based annotation, transcriptome reads were mapped onto the assembled genome using HISAT2 v0.1.6 (HISAT2, RRID:SCR_015530) [23]. Then, Cufflinks v2.2.1 (Cufflinks, RRID:SCR_014597) [24] was used to predict gene structures based on the transcriptome alignments. Finally, the gene sets from the 3 aforementioned approaches were merged to be a non-redundant and comprehensive gene set by MAKER v2.31.8 (MAKER Web Annotation Service, RRID:SCR_005318) [25]. A total of 44,086 protein-coding genes were predicted in the oriental river prawn genome (Table 1).

**Table 1:** Statistics of the genome assembly, Hi-C results, and gene set

Parameter	Scaffold	Contig
**Genome assembly and Hi-C result**		
Total No.	33,155	68,757
Total length (bp)	4,491,828,782	4,474,027,782
Average length (bp)	135,480	65,070
N50 Length (bp)	86,821,439	231,177
N90 Length (bp)	52,992,041	36,288
Maximum length (bp)	219,860,744	4,543,791
GC content (%)	36.95	36.95
**Gene annotation**		
Protein-coding gene No.		44,086
Mean transcript length (bp)		14,343
Mean exons per gene		5.0
Mean exon length (bp)		1,436.0
Mean intron length (bp)		3,034.0

The final gene set was functionally annotated by a BLAST-based analysis against SwissProt [26], TrEMBL [27], and KEGG [28] databases. In addition, InterProScan v4.7 (InterProScan, RRID:SCR_005829) [29] was used to search the translated protein sequences against other public databases, including Pfam [30], PRINTS [31], ProDom [32], and SMART [33], to determine known motifs and domains in our protein sequences.

### Evolutionary analysis

The reference protein sequences of 6 representative species (*D. melanogaster, D. pulex, Pinctada fucata, Cataglyphis savignyi, Litopenaeus vannamei*, and *Platyprepia virginalis*) were downloaded from NCBI. These protein sets and the oriental river prawn protein set were merged and filtered to remove those proteins <50 amino acids in length. All-to-all aligning was performed by using BLASTP v2.2.25 (BLASTP, RRID:SCR_001010) [21] (e-value ≤ 10^−5^) to identify homologous sequences. These proteins were then clustered into gene families by OrthoMCL (v2.09) [34]. As a result, it was determined that 444 single-copy orthologous gene families were shared by all 7 of the examined species.

To define the phylogenetic position of the oriental river prawn, we used MUSCLE v3.8.31 (MUSCLE, RRID:SCR_011812) [35] to align the single-copy orthologous genes. Then, the protein sequences were transformed to the corresponding nucleotide sequences, which were concatenated to a single “supergene” for each species. Alignments of these supergenes were carried out to construct a phylogenetic tree by using the maximum likelihood method in PhyML v3.0 (PhyML, RRID:SCR_014629) with the HKY85 model and default parameters [36]. Subsequently, the MCMCTREE program in the PAML package v4.8 [37] was used to predict divergence times with the assistance of fossil records from TIMETREE [38].

### 4dTv and genome duplication analyses

A 4-fold degenerative third-codon transversion (4dTv) analysis was performed to identify whole-genome duplication (WGD) of the oriental river prawn by comparing its genome with the published penaeid shrimp (*L. vannamei*) genome. Protein sequences from the 2 genomes were aligned using all-to-all BLASTp with an e-value of 1e−5. Subsequently, synteny blocks from oriental river prawn vs oriental river prawn, oriental river prawn vs penaeid shrimp, and penaeid shrimp vs penaeid shrimp were determined by MCscan v0.8 (MCscan, RRID:SCR_017650) [39] with default parameters. Homologous protein sequences from these syntenic regions were retrieved and converted to nucleotide sequences for alignments by MUSCLE v3.8.31 (MUSCLE, RRID:SCR_011812) [35]. Last, 4dTv values were predicted and corrected with the HKY model in the PAML package [37].

### Transcriptome and enrichment analyses

Raw transcriptome reads were filtered by removal of those reads with adaptor sequences, >10% of N bases, and >50% of low-quality bases (base quality score ≤10). These cleaned RNA reads were mapped onto the assembled genome of oriental river prawn using HISAT2 v0.1.6 (HISAT2, RRID:SCR_015530) with parameters “–phred33 –sensitive –no-discordant –no-mixed -I 1 -X 1000” [23]. Cufflink v2.2.1 (Cufflink, RRID:SCR_014597) with defaulted parameters was used to predict transcription values [24]. Cuffdiff in the Cufflink package with parameters of “-FDR 0.05–geometric-norm TRUE –c 10” was used to predict differentially expressed genes (DEGs) in the testis and the androgenic gland between reproductive and non-reproductive seasons. The edgeR software (edgeR, RRID:SCR_012802) [40] was used to draw heat maps with the threshold of *P*-value <0.05 and fold change >2. Finally, enriched Gene Ontology (GO) and KEGG terms were identified for these DEGs using the Enrich Pipeline as described previously [41].

## Results

### High-quality genome assembly and annotation

Approximately 293.3 Gb of Illumina reads and 405.7 Gb of PacBio long reads were sequenced. The genome assembly for the oriental river prawn spanned ∼4.5 Gb, with a contiguous N50 of 231.2 kb. The BUSCO v3.03 (BUSCO, RRID:SCR_015008) [42] value of this assembly was 92.6%, where C = 82.9%, F = 9.7%, M = 7.4%, and n = 1,066 (C: complete, F: fragmented, M: missing, and n: number of genes), suggesting a high level of completeness for this oriental river prawn assembly.

**Figure 2:**
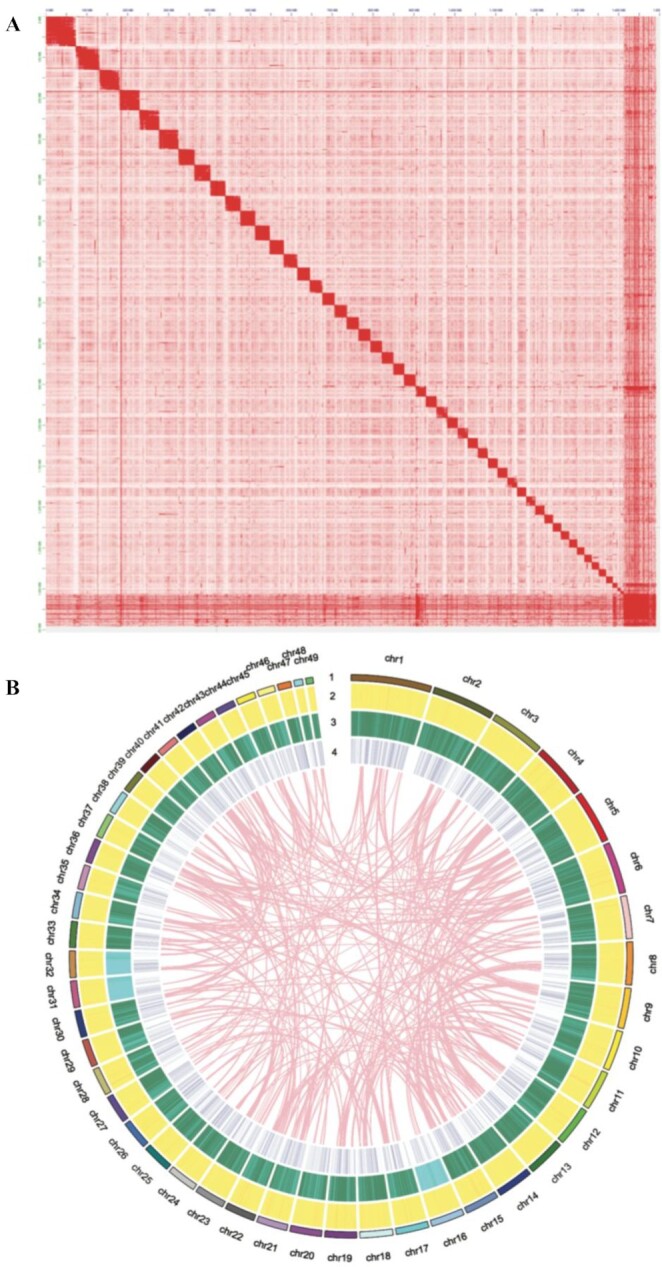
Hi-C interaction heat map and Circos view for the oriental river prawn genome. (A) A total of 49 chromosomes were constructed by Hi-C sequencing. (B) Circos view of the oriental river prawn. Layers include (1) chromosome length (Mb) and numbers; (2) distribution of gene density in 1-Mb non-overlapping windows; (3) distribution of repeat density in 1-Mb non-overlapping windows, with deeper green color indicating higher repeat density; and (4) distribution of GC content in 1-Mb non-overlapping windows, with pink lines representing inner synteny blocks.

Subsequently, a chromosome-level genome was assembled with an additional 876.4 Gb of Hi-C sequencing data [24]. Finally, 49 chromosomes were constructed with an anchored ratio of 94.7% (Fig. 2A) and a scaffold N50 of 86.8 Mb (Table 1). We also predicted 44,086 protein-coding genes, of which 39,317 genes have functional assignments with public databases. All distributions of genes, repeat sequences, and GC content are shown in Fig. 2B.

### Genome evolution and whole-genome duplication

**Figure 3:**
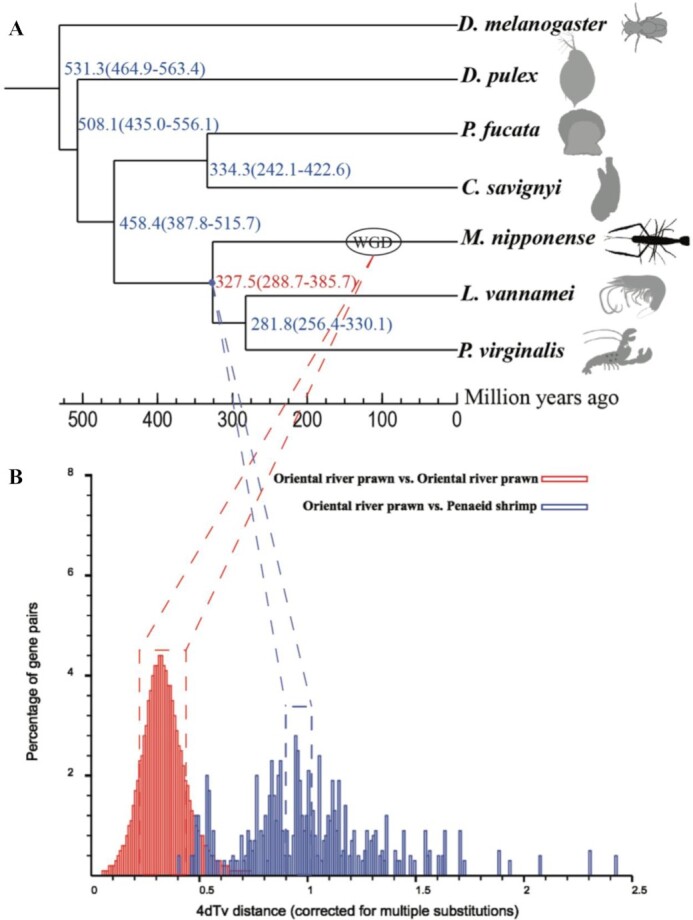
The divergence tree and the 4dTv results. (A) The divergence tree of 7 representative species. The numbers in parentheses represent confidence intervals. (B) The 4dTv distributions of oriental river prawn vs oriental river prawn (red) and oriental river prawn vs penaeid shrimp (blue).

After reconstruction of the phylogenetic and divergence trees, it was found that the oriental river prawn split from the last common ancestor of *L. vannamei* and *P. virginalis* ∼327.5 million years ago (Mya; Fig. 3A). Thirty-three synteny blocks were detected from penaeid shrimp self-alignment (penaeid shrimp vs penaeid shrimp). Conversely, 626,415 synteny blocks were discovered from oriental river prawn self-alignment (oriental river prawn vs oriental river prawn). The 4dTv analysis proposed a round of WGD in the oriental river prawn. After combining with the divergence time between penaeid shrimp and the oriental river prawn, we deduced that the WGD event happened ∼109.8 Mya (Fig. 3B).

### Sex divergence

**Figure 4:**
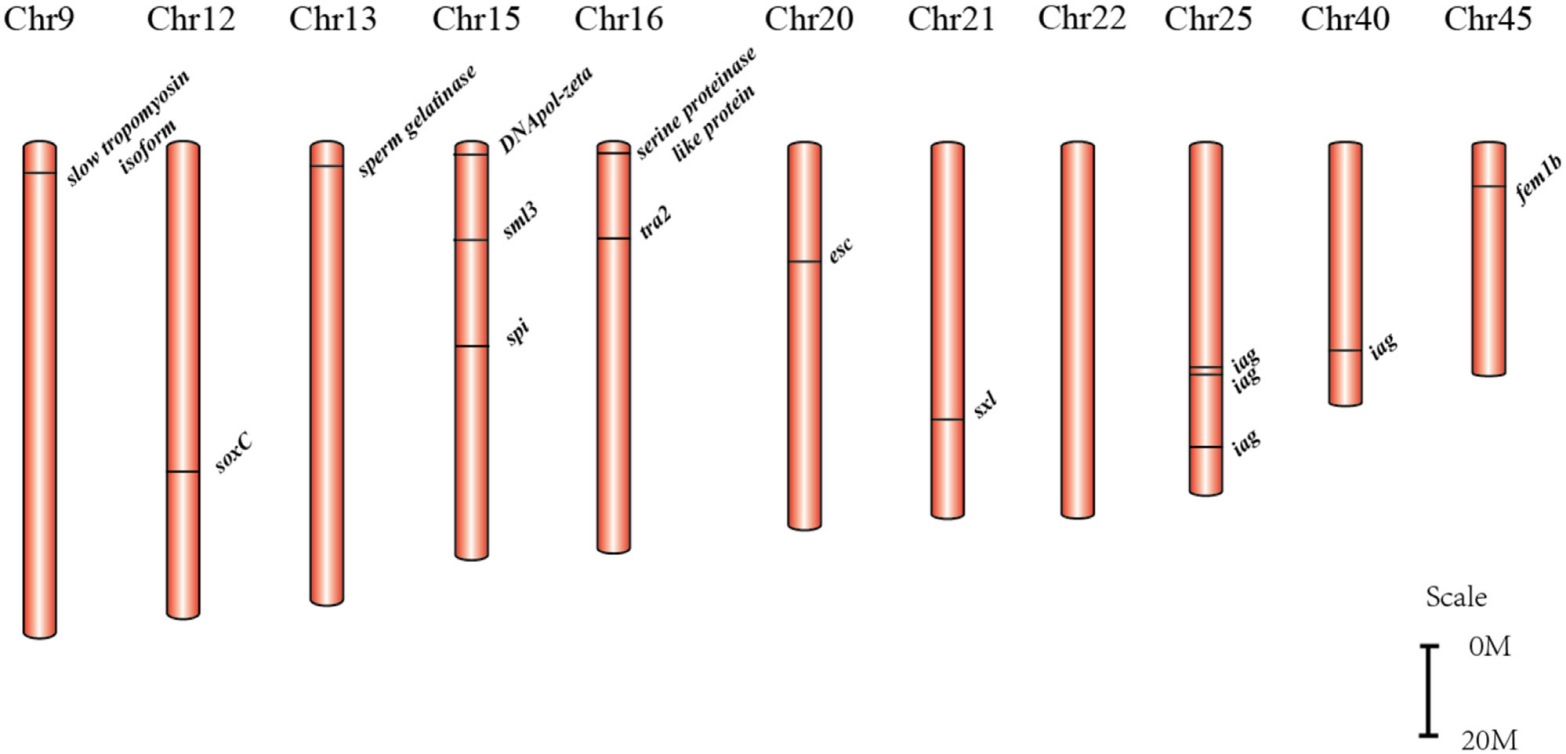
Sex-related candidate genes in the assembled oriental river prawn genome.

In previous studies [43–45], our research group identified 12 important genes for male sexual differentiation and development in the oriental river prawn, including insulin-like androgenic gland hormone (*iag*), sex-lethal (*sxl*), transformer-2 (*tra-2*), and extra sex comb (*esc*). We localized these sex-related genes on assembled chromosomes of the oriental river prawn, revealing a wide distribution on 11 chromosomes (Fig. 4).

Interestingly, 4 paralogous *iag* genes were identified throughout the whole genome, of which 3 were concentrated on chromosome (Chr) 25. The distance covering the 3 *iag* genes was 17.34 Mb, with prediction of 363 genes within this area. IAG, secreted by the androgenic gland, has been proved to function in male differentiation and development in various crustacean species [46–48]. A previous study [49] reported that injection of *iag* double-stranded RNA in giant freshwater prawns showed a significant inhibitory effect on male sexual differentiation and development of secondary sexual characteristics and spermatogenesis. On the basis of the important roles of *iag* in male sex determination and development of crustacean species [46–48], these genes on Chr25 were considered as strong candidate genes for sex differentiation and development in the oriental river prawn.

The androgenic gland and testis usually promote male sexual differentiation and testis development [50]. Many previous studies have determined that environmental factors can also dramatically affect the sexual differentiation and development processes, thereby leading to sex reversal. These environmental factors include temperature, illumination, and the presence of chemical pollutants [51]. Transcriptomic profiling analysis of the testis and androgenic glands between the reproductive season and the non-reproductive season were conducted, and a total of 309 genes were differentially expressed in testis, including 183 upregulated and 126 downregulated genes. A total of 238 DEGs were identified in the androgenic gland, including 146 upregulated and 92 downregulated genes. Among the 363 genes between *iag* genes on Chr25, 13 DEGs were, respectively, selected in testis and the androgenic gland, including 5 co-DEGs (Table 2). KEGG analysis revealed that these DEGs were involved in “Signal transduction,” “Endocrine system,” “Neurodegenerative diseases,” and “Lipid metabolism.”

**Table 2:** Statistics of important DEGs by comparing the testis and the androgenic gland transcriptome profiling between the reproductive and the non-reproductive seasons

DEGs	Reproductive season vs non-reproductive season (fold change)		Signaling pathways
	Testis	Androgenic gland	
Agrin	3.12	2.91	Signaling molecules and interaction
ETS homologous factor	2.56	3.13	Endocrine system
Glutamate receptor, ionotropic, kainate 2	1.78	2.41	Signal transduction; Endocrine system; Environmental adaptation; Lipid metabolism
Sodium- and chloride-dependent GABA transporter 3-like	0.65	0.56	
Glutamate receptor ionotropic	1.95	0.54	Endocrine system
Gamma-tubulin complex component 6	3.71		Signal transduction; Neurodegenerative diseases; Endocrine system
Histone regulator protein	1.97		
Peptidylprolyl isomerase F	2.67		Signal transduction; Neurodegenerative diseases
Peptidyl-prolyl cis-trans isomerase	2.16		Signal transduction; Neurodegenerative diseases
Protein gustavus isoform X1	2.36		
Aldose 1-epimerase-like	3.19		Signal transduction; Endocrine system; Lipid metabolism
7 transmembrane receptor	0.59		
Transcription factor protein	0.47		
NACHT, LRR, and PYD domains-containing protein 12-like	0.51		Infectious diseases: Bacterial; Infectious diseases: Viral; Immune system
Nesprin-1-like		3.16	
E3 ubiquitin-protein ligase TRIM32		4.19	Folding, sorting and degradation
Codanin-1-like		2.39	
Adhesion G protein-coupled receptor		0.29	
Myosin-IIIa		0.61	Transcription; Neurodegenerative diseases; Sensory system
Dynein assembly factor 5		0.54	
Histone-lysine N-methyltransferase SETMAR-like		0.49	

## Discussion

Karyotype analysis has been performed in many *Macrobrachium* species. A previous study [52] reported that the haploid number of chromosomes in oriental river prawn is 52. Other *Macrobrachium* species with reported karyotype analysis include the giant freshwater prawn (*Macrobrachium rosenbergii*; n = 59) [53], Dimua river prawn (*Macrobrachium villosimanus*; n = 62) [54], *Macrobrachium siwalikensis* (n = 50) [55], Lachester freshwater prawn (*Macrobrachium lachesteri*; n = 58) [56], freshwater shrimp (*Macrobrachium carcinus*; n = 47) [57], *Macrobrachium acanthurus* (n = 49) [57], and Amazon river prawn (*Macrobrachium amazonicum*; n = 49) [57]. In the present study, a total of 49 chromosomes were assembled, which is close to the reported number of haploid chromosomes in the oriental river prawn.

Here we have identified that the oriental river prawn had undergone a WGD event ∼109.8 Mya on the basis of large numbers of self-synteny blocks in this species. In a previous study [58], *Exopalaemon carinicauda*, a Palaemonidae species, was reported as not having genome duplication. According to our analysis of the *L. vannamei* genome (NCBI accession No. QCYY00000000), we identified that this species has not undergone the WGD event either. Therefore, this article is likely the first report of a recent WGD event in the *M. nipponense* genome.

Histological observations [59] demonstrated that the testis and androgenic gland of oriental river prawn in the non-reproductive vs reproductive season showed significant morphological differences. Therefore, those DEGs in the testis and androgenic gland between the non-reproductive vs reproductive seasons may participate in the male sexual differentiation and development processes in the oriental river prawn. We predicted in the present study that a total of 13 DEGs were respectively selected through transcriptomic profiling analysis (Table 2), of which 5 were co-DEGs. A few plausible sex-related candidate genes were identified, particularly after combining the analysis of genes on Chr25 and differential transcription in testis and androgenic gland between the non-reproductive and reproductive seasons. However, these results require more independent validation.

## Conclusions

A high-quality chromosome-level genome of oriental river prawn was assembled, by integration of Illumina, PacBio, and Hi-C sequencing. The whole-genome assembly was ∼4.5 Gb in size, with a contig N50 of 231.2 kb. A total of 49 chromosomes were generated with an anchored ratio of 94.7% and a scaffold N50 of 86.8 Mb. The oriental river prawn was found to have split from the common ancestor of *L. vannamei* and *P. virginalis* ∼327.5 Mya and to have undergone a WGD event that happened ∼109.8 Mya. Twenty-one sex-related candidate genes were identified after combining genome-wide screening and transcriptome profiling of testis and androgenic gland between the reproductive and non-reproductive seasons, although these results require further in-depth validation.

## Data Availability

The data that support the findings of this study have been deposited in CNGB Sequence Archive of China National GeneBank DataBase with accession No. CNP0001186. Genomic data are available via EBI bioproject IDs PRJNA646023 and PRJNA541743. Supporting data and materials are also available in the *GigaScience* GigaDB database [60].

## Abbreviations

4dTv: 4-fold degenerative third-codon transversion; AEP: aldose 1-epimerase protein; BLAST: Basic Local Alignment Search Tool; bp: base pairs; BUSCO: Benchmarking Universal Single-Copy Orthologs; BWA: Burrows-Wheeler Aligner; Chr: chromosome; DEG: differentially expressed gene; *esc*: extra sex comb; Gb: gigabase pairs; GC: guanine-cytosine; gDNA: genomic DNA; GO: Gene Ontology; Grik2: glutamate receptor: ionotropic: kainate 2; *iag*: insulin-like androgenic gland hormone; kb: kilobase pairs; KEGG: Kyoto Encyclopedia of Genes and Genomes; KO: KEGG Orthology; Mb; megabase pairs; Mya: million years ago; NCBI: National Center for Biotechnology Information; PacBio: Pacific Biosciences; PL: post-larval developmental stages; *sxl*: sex-lethal; *tra-2*: transformer-2; WGD: whole-genome duplication.

## Competing Interests

The authors declare that they have no competing interests.

## Funding

This research was supported by grants from the National Key R & D Program of China (2018YFD0901303); Special Scientific Research Funds for Central Non-profit Institutes, CAFS (2020TD36); Jiangsu Agricultural Industry Technology System (*Macrobrachium nipponense*); The Important New Varieties Selection Project of Jiangsu Province (PZCZ201745); China Agriculture Research System-48 (CARS-48); and Fund of Three Innovations Engineering of Jiangsu Province (D2015–16).

## Authors’ Contributions

H.F. and Q.S. conceived the project. S. Jiang and Y.X. collected and dissected the samples. K.H., C.S., and G.F. estimated genome size and assembled the genome. Z.G., R.L., Y.H., and X.Y. performed genome assembly, genome annotation, and evolution analysis. S. Jin, C.B., and H.Q. wrote the manuscript. W.Z. and Y.G. revised the manuscript.

## Supplementary Material

giaa160_GIGA-D-20-00274_Original_Submission

giaa160_GIGA-D-20-00274_Revision_1

giaa160_GIGA-D-20-00274_Revision_2

giaa160_Response_to_Reviewer_Comments_Original_Submission

giaa160_Response_to_Reviewer_Comments_Revision_1

giaa160_Reviewer_1_Report_Original_Submission Marco Gerdol -- 10/7/2020 Reviewed

giaa160_Reviewer_1_Report_Revision_1 Marco Gerdol -- 11/15/2020 Reviewed

giaa160_Reviewer_2_Report_Original_Submission Andrew Severin -- 10/6/2020 Reviewed
