# Supplementary material for: A chromosome-level genome assembly of the oriental river prawn, Macrobrachium nipponense
Source: Gigascience. 2021 Jan 18;10(1):giaa160. doi: 10.1093/gigascience/giaa160 (PMC7812440; doi:10.1093/gigascience/giaa160)
Supplement: giaa160_GIGA-D-20-00274_Revision_2 [file giaa160_giga-d-20-00274_revision_2.pdf]

GigaScience
A chromosome-level genome assembly of the Oriental river prawn, Macrobrachium nipponense
--Manuscript Draft--

Manuscript Number:	GIGA-D-20-00274R2
Full Title:	A chromosome-level genome assembly of the Oriental river prawn, Macrobrachium nipponense
Article Type:	Data Note
Funding Information:	
Abstract:	Background: The Oriental river prawn, Macrobrachium nipponense, is an economically important shrimp in China. Male prawns have higher commercial value than females because the former grow faster and reach larger sizes. It is therefore critical to reveal sex-differentiation and development mechanisms of the Oriental river prawn for genetic improvements.<br>Results: We sequenced 293.3 Gb of raw Illumina short reads and 405.7 Gb of Pacbio long reads. The final whole genome assembly of the Oriental river prawn was about 4.5 Gb, with predictions of 44,086 protein-coding genes. A total of 49 chromosomes were determined, with an anchor ratio of 94.7% and a scaffold N50 of 86.8 Mb. A whole genome duplication event was predicted to have happened 109.8 million years ago. By integration of genome and transcriptome data, 21 genes were predicted as sex-related candidate genes.<br>Conclusion: The first high-quality chromosome-level genome assembly of the Oriental river prawn was obtained. These genomic data, along with transcriptome sequences, are essential for understanding sex-differentiation and development mechanisms in the Oriental river prawn, as well as providing genetic resources for in-depth studies on developmental and evolutionary biology in arthropods.
Corresponding Author:	Shubo Jin<br>Chinese Academy of Fishery Sciences Freshwater Fisheries Research Center<br>Wuxi, Jiangsu CHINA
Corresponding Author Secondary Information:	
Corresponding Author's Institution:	Chinese Academy of Fishery Sciences Freshwater Fisheries Research Center
Corresponding Author's Secondary Institution:	
First Author:	Shubo Jin
First Author Secondary Information:	
Order of Authors:	Shubo Jin
	Chao Bian
	Sufei Jiang
	Kai Han
	Yiwei Xiong
	Wenyi Zhang
	Chengcheng Shi
	Hui Qiao
	Zijian Gao
	Ruihan Li
	Yu Huang
	Yongsheng Gong

Powered by Editorial Manager® and ProduXion Manager® from Aries Systems Corporation

	Xinxin You
	Guangyi Fan
	Qiong Shi
	Hongtuo Fu
**Order of Authors Secondary Information:**	
**Response to Reviewers:**	Reviewer #1: Thank you for providing a revised version of your MS along with detailed replies to my comments.<br>I find that most of my concerns have been adequately solved. However, a few additional modifications would be needed in my opinion.<br>I understand the rationale for using multiple individuals, and I appreciate the strategy of selecting multiple individuals originated from the same parents to minimize the issues linked with heterozygosity in the assembly. I have, however, a strong objection here, as k-mer-based estimates of genome size are not reliable under such circumstances, due the possible non-perfect overlap of the peaks generated by gDNA from multiple individuals, as the quantity of gDNA present in the pool from each individual are unlikely to be identical. I therefore strongly suggest the authors to remove the mention of k-mer based genome size estimates, as these would be only reliable with the analysis of Illumina reads generated from a single individual.<br>Reply: Thanks for your instructive comments. Sorry for the misleading descriptions in the previous version. In fact, the Illumina reads for our k-mer analysis was generated from a single individual. We extracted gDNAs from the muscle of this individual, and constructed two short libraries with the insert sizes of 500 and 800 bp respectively for the Illumina sequencing. The whole genome size was then estimated to be 4.6 Gb by the k-mer analysis.<br>However, we thought the gDNAs from a single individual would be insufficient for whole genome sequencing. Therefore, we collected five more individuals for Illumina sequencing and another batch of five individuals for Pacbio Sequencing, in order to provide sufficient gDNAs for the practical whole genome sequencing. Please find more details in the revised manuscript (lines 80-93).<br><br>Line 197: how was the HKY85 model selected? Did the authors use ModelTest to identify the best-fitting model of molecular evolution?<br>Reply: In our study, PhyML was employed to construct the phylogenetic tree with the default model HKY85. Thus, we tried to identify the best-fitting model to revise the phylogenetic tree by using the jModelTest. The best-fitting model was identified as GTR. Based on the GTR model, we rebuilt the phylogenetic tree, (Drosophila_melanogaster,(Daphnia_pulex,((Pinctada_fucata,Ciona_savignyi),(Macrobrachium_nipponense,(Procambarus_virginalis,Litopenaeus_vannamei))))). However, this revised tree has the same topology as the previous one. After fossil calibration of divergence times with the MCMCTREE model in PAML, we also observed that the revised tree has no difference from the previous one.<br><br>Figure 3a: please indicate what do the numbers indicated between brackets mean (confidence intervals, I guess)<br>Reply: Yes, you are right. The numbers within brackets represent confidence intervals. Thais sentence was added to the figure legend of Figure 3 (line 386).<br><br>Lines 298-300: please improve the English language used here.<br>Reply: Thanks for your advice. Yes, it is done in lines 304-307 of the manuscript.<br><br>The WGD discussion remains unconvincing. The authors mentioned Yuan et al. 2017 (the E. carinicauda genome) stating that this genome was not characterized by a WGD event. Actually, both genome size (5.7Gb) and 2n chromosome number (90) do not indicate significant differences compared with M. rosembergii. On the other hand, the number of genes is more than 2X higher in E. carinicauda. The quality of the E. carinicauda genome assembly was much lower than the genome reported in this study, so do the authors think that clues linked with WGD might have been missed in the study by Yuan et al.? Please also note that other Palaemonidae species have been previously shown to be characterized by very large genomes. For example Palemon serratus, with a c-value > 10. Overall, I believe that the timing of this WGD event cannot be defined with certainty at the present time due to the lack of genome and

Powered by Editorial Manager® and ProduXion Manager® from Aries Systems Corporation

	karyotype information for many species, so I would appreciate if the authors could state more explicitly that many uncertainties about this inference remain.<br>Reply: Thanks for your comments. Yes, you are right; we really think the important clues of WGD event have been missing in the Yuan's study, because the contig N50 of the E. carinicauda genome is only 263bp. These data are indeed fragmental to be used for gene identification. Therefore, too many genes and synteny blocks in the E. carinicauda genome were missed.<br>For the genome size, rich repeat content could be a major reason for the genome expansion. Based on our experience, the Palemon serratus could have much more repeat content.<br>For the WGD analysis, we employed both 4dtv and synteny block analyses that were well-performed and classical methods for WGD identification in various animal and plant genome reports. In the coming future, if there is any newly published genome assembly of a close shrimp species, we will further analyze related WGD event/time.<br>According to your advice, we rewrote related sentences. Please find more details in lines 301-307.
**Additional Information:**	
**Question**	**Response**
Are you submitting this manuscript to a special series or article collection?	No
**Experimental design and statistics**<br><br>Full details of the experimental design and statistical methods used should be given in the Methods section, as detailed in our Minimum Standards Reporting Checklist. Information essential to interpreting the data presented should be made available in the figure legends.<br><br>Have you included all the information requested in your manuscript?	Yes
**Resources**<br><br>A description of all resources used, including antibodies, cell lines, animals and software tools, with enough information to allow them to be uniquely identified, should be included in the Methods section. Authors are strongly encouraged to cite Research Resource Identifiers (RRIDs) for antibodies, model organisms and tools, where possible.<br><br>Have you included the information requested as detailed in our Minimum Standards Reporting Checklist?	Yes

**Availability of data and materials**<br><br>All datasets and code on which the conclusions of the paper rely must be either included in your submission or deposited in publicly available repositories (where available and ethically appropriate), referencing such data using a unique identifier in the references and in the "Availability of Data and Materials" section of your manuscript.<br><br>Have you have met the above requirement as detailed in our Minimum Standards Reporting Checklist?	Yes

Manuscript
Click here to access/download;Manuscript;Manuscript 11-25 revised-HL.docx

A chromosome-level genome assembly of the Oriental river prawn, *Macrobrachium nipponense*

Shubo Jin [1,†], Chao Bian [2,†], Sufei Jiang [1,†], Kai Han [3], Yiwei Xiong [1], Wenyi Zhang [1], Chengcheng Shi [3], Hui Qiao [1], Zijian Gao [2], Ruihan Li [2], Yu Huang [2], Yongsheng Gong [1], Xinxin You [2], Guangyi Fan [3], Qiong Shi [2*], Hongtuo Fu [1*]

† These authors contributed equally to this work.

1. Key Laboratory of Freshwater Fisheries and Germplasm Resources Utilization, Ministry of Agriculture, Freshwater Fisheries Research Center, Chinese Academy of Fishery Sciences, Wuxi 214081, China;

2. Shenzhen Key Lab of Marine Genomics, Guangdong Provincial Key Lab of Molecular Breeding in Marine Economic Animals, BGI Academy of Marine Sciences, BGI Marine, BGI, Shenzhen 518083, China;

3. BGI-Qingdao, BGI-Shenzhen, Qingdao, 266555, China.

[†]Contributed equally to this work.

*Corresponding authors.

∗Correspondence address. Hongtuo Fu, Freshwater Fisheries Research Center, Chinese Academy of Fishery Sciences, Wuxi, Jiangsu 214081, China. Tel: +86-136 01480163; E-mail: fuht@ffrc.cn; http://orcid.org/0000-0002-6550-9268; Qiong Shi, BGI Academy of Marine Sciences, BGI Marine, BGI, Shenzhen, Guangdong 518083, China. Tel: +86-185 6627 9826; E-mail: shiqiong@genomics.cn; http://orcid.org/0000-0001-7007-8530

ORCIDs:

Shubo Jin, 0000-0002-6550-9268;

Chao Bian, 0000-0001-9904-721x;

Sufei Jiang, 0000-0002-4745-8639;

Qiong Shi, 0000-0002-6358-976x;

Hongtuo Fu, 0000-0003-2974-9464.

Abstract

Background: The Oriental river prawn, *Macrobrachium nipponense*, is an economically important shrimp in China. Male prawns have higher commercial value than females because the former grow faster and reach larger sizes. It is therefore important to reveal sex-differentiation and development mechanisms of the Oriental river prawn to enable genetic

improvement.
Results: We sequenced 293.3 Gb of raw Illumina short reads and 405.7 Gb of Pacbio long reads. The final whole genome assembly of the Oriental river prawn was about 4.5 Gb in size, with predictions of 44,086 protein-coding genes. A total of 49 chromosomes were determined, with an anchor ratio of 94.7% and a scaffold N50 of 86.8 Mb. A whole genome duplication event was predicted to have happened 109.8 million years ago. By integration of genome and transcriptome data, 21 genes were predicted as sex-related candidate genes.
Conclusion: The first high-quality chromosome-level genome assembly of the Oriental river prawn was obtained. These genomic data, along with transcriptome sequences, are essential for understanding sex-differentiation and development mechanisms in the Oriental river prawn, as well as providing genetic resources for in-depth studies on developmental and evolutionary biology in arthropods.

Introduction

The Oriental river prawn, *Macrobrachium nipponense* (NCBI:txid159736; marinespecies.org:taxname:587405; Subphylum Crustacea, Order Decapoda, Family Palaemonidae; Figure 1), is widely distributed in freshwater and low-salinity estuarine regions of China [1, 2]. It has become an important commercial species in China due to its high nutritional value and delicious taste. Its annual production has gradually increased in recent years (up to 272,592 tons in 2016) (Bureau of Fisheries, Ministry of Agriculture, P. R. China, 2016). The annual output value was approximately 2.8 billion US dollars.

It is notable that growth performance of the Oriental river prawn shows remarkable differences between male and female individuals. Males usually grow faster than their female counterparts and reach larger sizes at the harvest period each year. Thus, culturing all-male populations is a long-term goal for practical aquaculture, and will lead to a dramatic increase in economic profits. In addition, our previous study [3] has reported that both ovaries and testis in Oriental river prawns begin to differentiate at a post-developmental stage (day 13; PL13), and mature at PL19 to PL22, based on histological observations and steroid hormone levels. In practice, quick gonad development restricts sustainable growth of the Oriental river prawn industry, because over-reproduction will happen frequently during the farming process, leading to poor survival, low growth rates, and small body size. Therefore, it is critical to understand the mechanism of sexual differentiation and reproductive development in Oriental river prawn in order to obtain genetic improvement.

In our present study, a chromosome-level genome assembly for the Oriental river prawn was constructed by integration of PacBio long reads, Illumina short reads, and Hi-C sequencing data. These genomic data along with transcriptome sequences will be a useful resource for in-depth studies on sex-differentiation and the mechanism of reproduction in Oriental river prawn, as well as promoting comparative genomic analyses with other prawn species.

Methods

Sample collection

Specimens of Oriental river prawn were collected from a wild population in Tai Lake, Wuxi, China (120°13′44″E, 31°28′ 22″N). In a lineage family, one individual with the body weight of 14.01 g was selected for a k-mer analysis; five individuals with body weights of 13.02–15.56 g were selected for Illumina sequencing, and another five individuals with body weights of 10.50–13.06 g were selected for PacBio sequencing. Fresh muscle tissues of multiple individuals from both groups were collected respectively and immediately frozen in liquid nitrogen before carrying out DNA extraction.

DNA extraction and whole genome sequencing

Muscle tissues from the five individuals in each group were pooled, and then genomic DNAs (gDNAs) from the pooled samples were extracted using a Nucleic Acid Kit (Qiagen, Germantown, MD, USA) in accordance with the manufacturer's instructions. gDNAs were also extracted from the muscle of a single individual for the k-mer analysis. The extracted gDNAs was then used for constructing libraries for Illumina (Illumina Inc., San Diego, CA, USA) and PacBio (Menlo Park, CA, USA) sequencing. According to the Illumina's protocols, two short paired-end libraries (with the insert sizes of 500 and 800 bp, respectively) were constructed for the k-mer analysis. Another seven paired-end libraries (with the insert sizes of 270 bp, 500 bp, 800 bp, 2 kb, 5 kb, 10 kb and 20 kb, respectively) were constructed for the whole genome sequencing. These libraries were then sequenced on an Illumina HiSeq X-Ten platform (PE150 in length; RRID:SCR_016385). About 157.3 Gb of raw-reads were generated for the k-mer analysis, and approximately 293.3 Gb of raw sequenced reads were produced for the whole genome assembly. Subsequently, 253.4 Gb of clean data were retained for assembly through filtering low-quality data and removal of duplicated reads and adapter sequences by SOAPfilter v2.2 (RRID: SCR_014986)[4]. In parallel to this, long inserted libraries were created using the PacBio Sequel platform (RRID:SCR_017989) for the whole genome assembly. About 405.7 Gb of PacBio long reads were generated. These long reads were then corrected by LoRDEC

(RRID:SCR_015814) [5] using default parameters.

RNA extraction and transcriptome sequencing

Male individuals of Oriental river prawn in both reproductive and non-reproductive seasons were also collected from a wild population in Tai Lake, Wuxi, China. Shrimp in the non-reproductive season with body weights of 2.54–5.08 g were collected in January 2018 (water temperature ≤ 15 °C, light cycle ≤ 10 h), while shrimps in the reproductive season with body weights of 3.07–5.24 g were collected in July 2018 (water temperature of ≥ 28 °C, light cycle ≥ 16 h).

All the prawns were transferred to a 500-L indoor tank with aerated freshwater for 2 days before tissue collection. Testes and androgenic glands were collected from specimens in the non-reproductive season and reproductive season. At least 0.5 g of testes and androgenic glands (n > 50) were pooled to form one biological replicate, and three biological replicates were separated for transcriptome sequencing. These collected samples were immediately frozen in liquid nitrogen and stored at −80 °C until use.

Total RNA was extracted using a UNlQ-10 Column Trizol Total RNA Isolation Kit (Sangon Biotech, Shanghai, China) following the manufacturer's protocol. In brief, the total mRNAs were digested individually into fragments, and 200-bp raw paired-end reads were generated using the Illumina sequencing platform.

Hi-C library preparation

To construct pseudo-chromosomes, anther ten male individuals of Oriental river prawn with body weights of 10.16-13.45_g were collected from the same lineage. Blood samples of these individuals were also collected. A Hi-C library was constructed with the pool of extracted blood gDNAs, and it was then sequenced on an Illumina HiSeq X-Ten platform.

Genome assembly

The genome size of the Oriental river prawn was estimated by using a routine 17-mer frequency distribution analysis [6] on cleaned Hiseq data with insert sizes of 500 bp and 800 bp. The genome size was calculated according to the following equation: genome size=k-mer number/the expected k-mer depth. In the case of sufficient data, the k-mer frequency distribution follows a Poisson distribution pattern, and the peak of the k-mer distribution curve is considered as the expectation of k-mer depth. As a result, the genome size of the Oriental river prawn was estimated to be approximately 4.6 Gb.

Long reads sequenced using the PacBio platform were assembled by using the Shasta long read assembler v0.2.0 [7] with 200 minimal component size ('--ReadGraph.min ComponentSize') to ensure that the best quality read graph and 50 minimal aligned markers ('--Align.min AlignedMarkerCount') matched aligned read pairs. The consensus caller model was set as 'Modal' to assemble repeat counts. Those paired-end reads with an insert size of 200–800 bp were aligned against the draft assembly using BWA (v0.7.12, RRID: SCR_010910) [8], and the assembled sequences were then improved through two rounds of polishing using Pilon (v1.23, RRID: SCR_014731) [9] based on the read alignments.

To improve the draft genome assembly to a chromosome level, a Hi-C library was constructed following the Rao's method [10] using pooled blood gDNAs. Chromatins were cross-linked with formaldehyde and digested with MboI enzyme; subsequently, the generated sticky ends were filled and further ligated, and then DNAs were purified and sheared. Paired-end sequencing was performed using the BGI-seq 500 platform (BGI, Shenzhen, China). First, all valid read pairs were extracted based on the results of Hic-Pro (v2.8.0, RRID: SCR_017643) [11] and further aligned to the draft genome assembly using Juicer (v1.5, RRID: SCR_017226) [12] to generate the Hi-C interaction maps ('merged_nodups.txt' file). Subsequently, scaffold sequences from the draft assembly were ordered and oriented using the 3D-DNA pipeline [13] to integrated into long pseudo-chromosomes. Manual review and refinement were performed for identification and removal of assembly errors with assistance of the Juicebox Assembly Tool v1.9.0 [14].

Repeat and gene structure annotation

Two routine approaches, including *ab initio* and homology prediction methods, were used to detect repetitive elements in the genome assembly. In the *ab initio* prediction, the RepeatModeler (v1.04, RRID:SCR_015027) [15] and LTR-FINDER (v1.06, RRID:SCR_015247) [16] were used with default parameters to detect repetitive elements. Then, a *de novo* repeat sequence library was built by using above results. Subsequently, RepeatMasker (RRID:SCR_012954) [17] was used to annotate the novel library based on Repbase TE (v14.04) [18]. Additionally, Tandem Repeats Finder (v4.04) [19] was applied to identify the tandem elements. For the homology prediction, RepeatMasker [17] and RepeatProteinMask (v3.2.2) [17] were used to search the repeat elements among the assembled genome based on RepBase TE (v14.04) [18]. After combining the results from above-mentioned two approaches, it was found that repeat sequences accounted for about 50.2% of the assembled genome. Finally, repeat regions were masked in the genome of the Oriental river

prawn for prediction of protein-coding genes.

An integration of three methods, including *de novo* prediction, homology-based annotation and transcriptome-based annotation, was applied to predict protein-coding genes in the assembled genome. For the *de novo* prediction, Augustus (v3.0.2, RRID:SCR 008417) [20] was performed to identify coding regions on the repeat-masked assembly with default parameters. For the homology-based prediction, protein sequences of nine representative species (*Caenorhabditis elegans, Eriocheir sinensis, Danio rerio, Daphnia pulex, Drosophila melanogaster, Homo sapiens, Crassostrea gigas, Pinctada fucata martensii*, and *Litopenaeus vannamei*) downloaded from the National Center for Biotechnology Information (NCBI) database were mapped onto the Oriental river prawn genome using TBLASTn (v2.2.25, RRID:SCR 001010) [21] with an e-value $\leq 10^{-5}$. Subsequently, GeneWise (v2.2.0, RRID:SCR_015054) [22] was applied to identify gene structures based on the best TBLASTn alignments. For the transcriptome-based annotation, transcriptome reads were mapped onto the assembled genome using HISAT2 (v0.1.6, RRID:SCR_015530) [23]. Then, Cufflinks (v 2.2.1, RRID:SCR_014597) [24] was used to predict gene structures based on the transcriptome alignments. Finally, the gene sets from the three above-mentioned approaches were merged to be a non-redundant and comprehensive gene set by MAKER (v2.31.8, RRID:SCR 005318) [25]. A total of 44,086 protein-coding genes were predicted in the Oriental river prawn genome (Table 1).

The final gene set was functionally annotated by a BLAST-based analysis against SwissProt [26], TrEMBL [27] and KEGG (Kyoto Encyclopedia of Genes and Genomes) [28] databases. In addition, the Inter-ProScan (version 4.7, RRID:SCR 005829) [29] was used to search the translated protein sequences against other public databases, including Pfam [30], PRINTS [31], ProDom [32], and SMART [33], in order to determine known motifs and domains in our protein sequences.

Evolutionary analysis

The reference protein sequences of six representative species (*D. melanogaster, D. pulex, Pinctada fucata, Cataglyphis savignyi, Litopenaeus vannamei,* and *Platyprepia virginalis*) were downloaded from NCBI. These protein sets and the Oriental river prawn protein set were merged and filtered to remove those proteins less than 50 amino acids in length. All-to-all aligning was performed by using BLASTP (v2.2.25, RRID:SCR 001010) [21] (e-value $\leq 10^{-5}$) to identify homologous sequences. These proteins were then clustered into gene families by OrthoMCL (v2.09) [34]. As a result, it was determined that 444 single-copy orthologous gene

families were shared by all of the examined seven species.

To define the phylogenetic position of the Oriental river prawn, we used MUSCLE (v3.8.31, RRID:SCR_011812) [35] to align the single-copy orthologous genes. Then, the protein sequences were transformed to the corresponding nucleotide sequences, which were concatenated to a single 'supergene' for each species. Alignments of these 'supergenes' were carried out to construct a phylogenetic tree by using the Maximum Likelihood method in PhyML (v3.0, RRID:SCR_014629) with the HKY85 model and default parameters [36]. Subsequently, the MCMCTREE program in the PAML package v4.8 [37] was employed to predict divergence times with assistance of fossil records from TIMETREE (http://www.timetree.org).

4dTv and genome duplication analyses

A 4-fold degenerative third-codon transversion (4dTv) analysis was performed to identify whole genome duplication (WGD) of the Oriental river prawn by comparing its genome with the published penaeid shrimp (*L. vannamei*) genome. Protein sequences from the two genomes were aligned using all-to-all BLASTp with an e-value of 1e-5. Subsequently, synteny blocks from Oriental river prawn vs. Oriental river prawn, Oriental river prawn vs. penaeid shrimp, and penaeid shrimp vs. penaeid shrimp were determined by MCscan (v0.8, RRID:SCR_017650) [38] with default parameters. Homologous protein sequences from these syntenic regions were retrieved and converted to nucleotide sequences for alignments by MUSCLE (v3.8.31, RRID:SCR_011812) [35]. Lastly, 4dTv values were predicted and corrected with the HKY model in PAML package [37].

Transcriptome and enrichment analyses

Raw transcriptome reads were filtered by removal of those reads with adaptor sequences, >10% of N bases, and >50% of low-quality bases (base quality score ≤ 10). These cleaned RNA reads were mapped onto the assembled genome of Oriental river prawn using HISAT2 (v0.1.6, RRID:SCR 015530) with parameters "–phred33 –sensitive –no-discordant –no-mixed -I 1 -X 1000" [23]. The Cufflink (v2.2.1, RRID:SCR 014597) with defaulted parameters was employed to predict transcription values [24]. The Cuffdiff in the Cufflink package with parameters of "-FDR 0.05 – geometric-norm TRUE –c 10" was utilized to predict differentially expressed genes (DEGs) in the testis and the androgenic gland between reproductive and non-reproductive seasons. The edgeR software (RRID:SCR 012802) [39] was used to draw heat maps with the threshold of p-value < 0.05 and fold change > 2. Finally, enriched Gene Ontology

(GO) and Kyoto Encyclopedia of Genes and Genomes (KEGG) terms were identified for these DEGs using the Enrich Pipeline as described previously [40].

Results

High-quality genome assembly and annotation

Approximately 293.3 Gb of Illumina reads and 405.7 Gb of PacBio long reads were sequenced. The genome assembly for the Oriental river prawn spanned ~4.5 Gb, with a contiguous N50 of 231.2 kb. The BUSCO (Benchmarking Universal Single-Copy Orthologs; University of Geneva Medical School and Swiss Institute of Bioinformatics, Geneva, Switzerland; v3.03, RRID:SCR_015008) [41] value of this assembly was 92.6%, where C = 82.9%, F = 9.7%, M = 7.4%, and n = 1066 (C: complete, F: fragmented, M: missing, and n: number of genes), suggesting a high level of completeness for this Oriental river prawn assembly.

Subsequently, a chromosome-level genome was assembled with an additional 876.4 Gb of Hi-C sequencing data [24]. Finally, 49 chromosomes were constructed with an anchored ratio of 94.7% (Figure 2a) and a scaffold N50 of 86.8 Mb (Table 1). We also predicted 44,086 protein-coding genes, of which 39,317 genes have functional assignments with public databases. All distributions of genes, repeat sequences and GC content were shown in Figure 2b.

Genome evolution and whole genome duplication

After reconstruction of the phylogenetic and divergence trees, it was found that the Oriental river prawn has split from the last common ancestor of *L. vannamei* and *P. virginalis* - approximately 327.5 million years ago (Mya; Figure 3a). Thirty-three synteny blocks were detected from penaeid shrimp self-aligning (penaeid shrimp vs. penaeid shrimp). Conversely, 626,415 synteny blocks were discovered from Oriental river prawn self-aligning (Oriental river prawn vs. Oriental river prawn). The 4dTv analysis proposed a round of WGD in the Oriental river prawn. After combing with the divergence time between penaeid shrimp and the Oriental river prawn, we predicted that the WGD event happened ~109.8 Mya (Figure 3b).

Sex divergence

In previous studies [42-44], our research group identified 12 important genes for male sexual differentiation and development in the Oriental river prawn, including insulin-like androgenic gland hormone (*iag*), sex-lethal (*sxl*), transformer-2 (*tra-2*) and extra sex comb (*esc*). We

localized these sex-related genes on assembled chromosomes of the Oriental river prawn, revealing a wide distribution on ten chromosomes (Figure 4).

Interestingly, four paralogous *iag* genes were identified throughout the whole genome, of which three were concentrated on the Chromosome (Chr) 25. The distance covering the three *iag* genes was 17.34 Mb with prediction of 363 genes within this area. IAG, secreted by the androgenic gland, has been proved to function in male differentiation and development in various crustacean species [45-47]. A previous study [48] reported that injection of *iag* dsRNA in giant freshwater prawns showed a significant inhibitory effect on male sexual differentiation and development of secondary sexual characteristics and spermatogenesis. Based on the important roles of *iag* in male sex-determination and development of crustacean species [45-47], these genes on the Chr25 were considered as strong candidate genes for sex-differentiation and development in the Oriental river prawn.

Androgenic gland and testis usually promote male sexual differentiation and testis development [49]. Many previous studies have determined that environmental factors can also dramatically affect the sexual differentiation and development processes, thereby leading to sex reversal. These environmental factors include temperature, illumination, and presence of chemical pollutants [50]. Transcriptomic profiling analysis of the testis and androgenic glands between the reproductive season and the non-reproductive season were conducted, and a total of 309 genes were differentially expressed in testis, including 183 up-regulated and 126 down-regulated genes. A total of 238 DEGs were identified in the androgenic gland, including 146 up-regulated and 92 down-regulated genes. Among the 363 genes between *iag* genes on the Chr25, 13 DEGs were respectively selected in testis and the androgenic gland, including five co-DEGs (Table 2). KEGG analysis revealed that these DEGs were involved in “Signal transduction”, “Endocrine system”, “Neurodegenerative diseases”, and “Lipid metabolism”.

Discussion

Karyotype analysis has been performed in many *Macrobrachium* species. A previous study [51] reported that the haploid number of chromosomes in Oriental river prawn is 52. Other *Macrobrachium* species with reported karyotype analysis include the giant freshwater prawn (*Macrobrachium rosenbergii*; n=59) [52], Dimua river prawn (*M. villosimanus*; n=62) [53], *M. siwalikensis* (n=50) [54], Lachester freshwater prawn (*M. lachesteri*; n=58) [55], freshwater shrimp (*M. carcinus*; n=47) [56], *M. acanthurus* (n=49) [56], and Amazon river prawn (*M. amazonicum*; n=49) [56]. In our present study, a total of 49 chromosomes were assembled, which is close to the reported number of haploid chromosomes in the Oriental river prawn.

Here we have identified that the oriental river prawn had undergone a WGD event about 109.8 Mya based on large numbers of self-synteny blocks in this species. In a previous study [57], *Exopalaemon carinicauda*, a Palaemonidae species, was reported as not having genome duplication. According to our analysis of the *L. vannamei* genome (NCBI accession number QCYY00000000), we identified that this species has not undergone the WGD event either. Therefore, this paper is likely the first report of a recent WGD event in the *M. nipponense* genome.

Histological observations [58] demonstrated that the testis and androgenic gland of Oriental river prawn in the non-reproductive vs. reproductive season showed significant morphological differences. Therefore, those DEGs in the testis and androgenic gland between the non-reproductive vs. reproductive seasons may participate in the male sexual differentiation and development processes in the Oriental river prawn. We predicted in the present study that a total of 13 DEGs were respectively selected through transcriptomic profiling analysis (Table 2), of which five were co-DEGs. A few plausible sex-related candidate genes were identified, particularly after combining the analysis of genes on Chromosome 25 and differential transcription in testis and androgenic gland between the non-reproductive and reproductive seasons. However, these results require more independent validation.

Conclusions

A high-quality chromosome-level genome of Oriental river prawn was assembled, by integration of Illumina, Pacbio and Hi-C sequencing. The whole genome assembly was ~4.5 Gb in size, with a contig N50 of 231.2 kb. A total of 49 chromosomes were generated with an anchored ratio of 94.7% and a scaffold N50 of 86.8 Mb. The Oriental river prawn was found to have split from the common ancestor of *L. vannamei* and *P. virginalis* ~327.5 Mya, and undergoing a WGD event that happened ~109.8 Mya. Twenty-one sex-related candidate genes were identified after combining genome-wide screening and transcriptome profiling of testis and androgenic gland between the reproductive and non-reproductive seasons, although these results require further in-depth validation.

Abbreviations

DEG, differentially expressed gene; KO, KEGG Orthology; 4dTv, 4-fold degenerative third-codon transversion; DEGs, differentially expressed genes; *iag*, insulin-like androgenic gland hormone; NCBI, National Center for Biotechnology Information; GO, Gene Ontology; KEGG, Kyoto Encyclopedia of Genes and Genomes; *sxl*, sex-lethal; *tra-2*, transformer-2; *esc*, extra

sex comb; Grik2: Glutamate receptor, ionotropic, kainate 2; AEP Aldose 1-epimerase protein; Chr, chromosome; Mya, million years ago; PL, post-larval developmental stages; WGD, whole genome duplication.

Availability of Supporting Data and Materials

The data that support the findings of this study have been deposited in CNGB Sequence Archive of China National GeneBank DataBase with accession number CNP0001186. Genomic data is available via EBI bioproject IDs PRJNA646023 and PRJNA541743. Supporting data and materials are also available in the *GigaScience* GigaDB database [59].

Acknowledgement

This research was supported by grants from the National Key R & D Program of China (2018YFD0901303); Special Scientific Research Funds for Central Non-profit Institutes, CAFS (2020TD36); Jiangsu Agricultural Industry Technology System (*Macrobrachium nipponense*); The Important New Varieties Selection Project of Jiangsu Province (PZCZ201745); China Agriculture Research System-48 (CARS-48); Fund of Three Innovations Engineering of Jiangsu Province (D2015–16).

Authors' Contributions

H.F. and Q.S. conceived the project. S.J. and Y.X. collected and dissected the samples. K.H., C.S. and G.F. estimated genome size and assembled the genome. Z.G., R.L., Y.H. and X.Y. performed genome assembly, genome annotation, and evolution analysis. S.J., C.B., and H.Q. wrote the manuscript. W.Z. and Y.G. revised the manuscript.

Table 1. Statistics of the genome assembly, Hi-C results and gene set.

**Genome assembly and Hi-C result**		
**Parameter**	**Scaffold**	**Contig**
Total Number	33,155	68,757
Total length (bp)	4,491,828,782	4,474,027,782
Average Length (bp)	135,480	65,070
N50 Length (bp)	86,821,439	231,177
N90 Length (bp)	52,992,041	36,288
Maximum Length (bp)	219,860,744	4,543,791

GC content	36.95%	36.95%
**Gene annotation**		
Protein-coding gene number		44,086
Mean transcript length (bp)		14,343
Mean exons per gene		5.0
Mean exon length (bp)		1,436.0
Mean intron length (bp)		3,034.0

Table 2. Statistics of important DEGs by comparing the testis and the androgenic gland transcriptome profiling between the reproductive and the non-reproductive seasons

DEGs	Testis<br>Reproductive season vs non-reproductive season<br>(Fold change)	Androgenic gland<br>Reproductive season vs non-reproductive season<br>(Fold change)	Signaling pathways
agrin	3.12	2.91	Signaling molecules and interaction
ETS homologous factor	2.56	3.13	Endocrine system
glutamate receptor, ionotropic, kainate 2	1.78	2.41	Signal transduction;<br>Endocrine system;<br>Environmental adaptation;<br>Lipid metabolism
sodium- and chloride-dependent GABA transporter 3-like	0.65	0.56	
glutamate receptor ionotropic	1.95	0.54	Endocrine system
gamma-tubulin complex component 6	3.71		Signal transduction;<br>Neurodegenerative diseases;<br>Endocrine system
protein HIRA	1.97		
peptidylprolyl isomerase F	2.67		Signal transduction;<br>Neurodegenerative diseases
peptidyl-prolyl cis-trans isomerase	2.16		Signal transduction;<br>Neurodegenerative diseases
protein gustavus isoform X1	2.36		
aldose 1-epimerase-like	3.19		Signal transduction;<br>Endocrine system;

			Lipid metabolism
7 transmembrane receptor	0.59		
transcription factor protein	0.47		
NACHT, LRR and PYD domains-containing protein 12-like	0.51		Infectious diseases: Bacterial;<br>Infectious diseases: Viral;<br>Immune system
nesprin-1-like		3.16	
E3 ubiquitin-protein ligase TRIM32		4.19	Folding, sorting and degradation
codanin-1-like		2.39	
adhesion G protein-coupled receptor		0.29	
myosin-IIIa		0.61	Transcription;<br>Neurodegenerative diseases;<br>Sensory system
dynein assembly factor 5		0.54	
histone-lysine N-methyltransferase SETMAR-like		0.49	

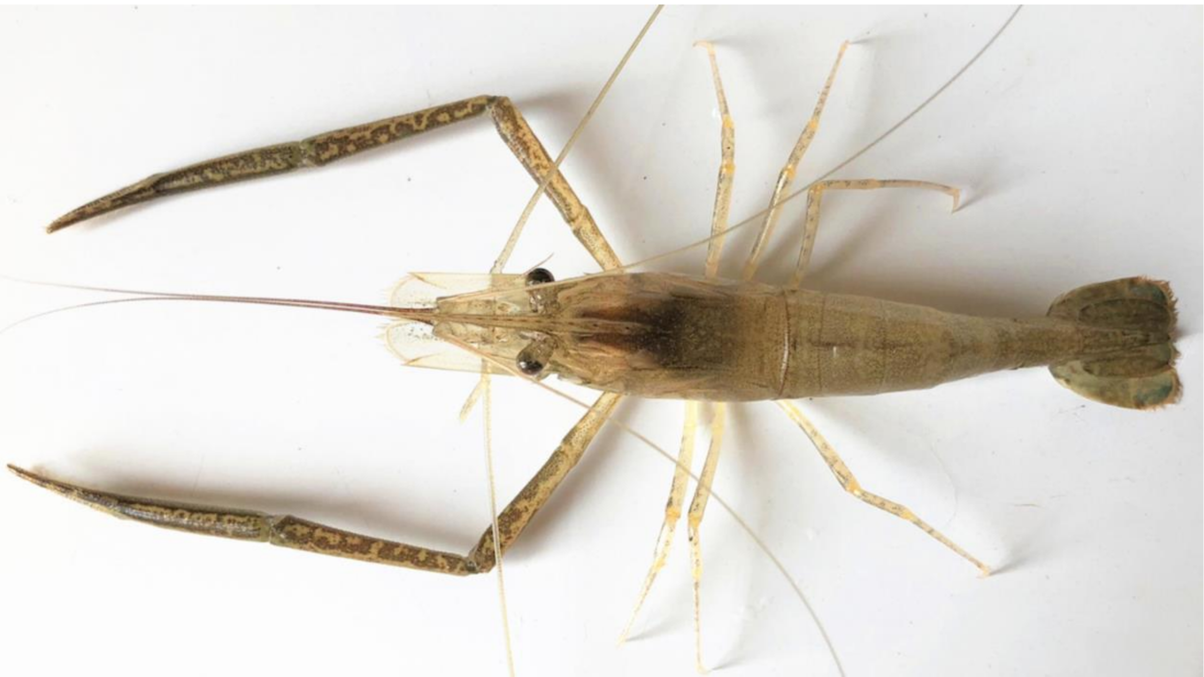

Figure 1. Picture of the sequenced oriental river prawn. This individual was collected from Taihu Lake, Wuxi City, Jiangsu Province, China.

a

b

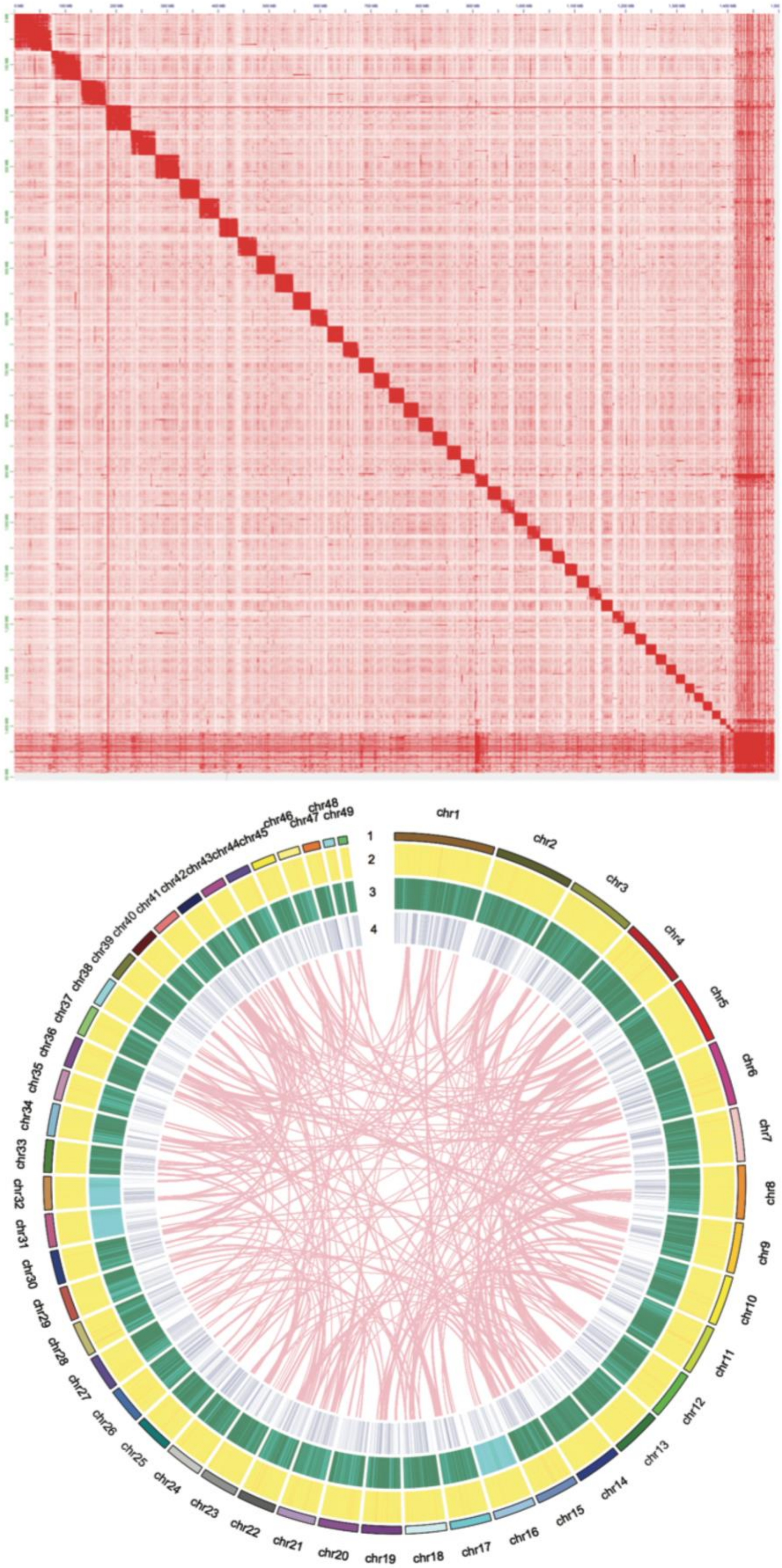
1
2
3
4
chr1
chr2
chr3
chr4
chr5
chr6
chr7
chr8
chr9
chr10
chr11
chr12
chr13
chr14
chr15
chr16
chr17
chr18
chr19
chr20
chr21
chr22
chr23
chr24
chr25
chr26
chr27
chr28
chr29
chr30
chr31
chr32
chr33
chr34
chr35
chr36
chr37
chr38
chr39
chr40
chr41
chr42
chr43
chr44
chr45
chr46
chr47
chr48
chr49

Figure 2. Hi-C interaction heat map and circos view for the oriental river prawn genome. (a) A total of 49 chromosomes were constructed by Hi-C sequencing. (b) circos view of the oriental river prawn. 1. Chromosome length (Mb) and numbers. 2. Distribution of gene density in 1Mb non-overlapping windows. 3. Distribution of repeat density in 1Mb non-overlapping windows. Deeper green color indicates higher repeat density. 4. Distribution of GC content in 1Mb non-overlapping windows. The pink lines represent the inner synteny blocks.

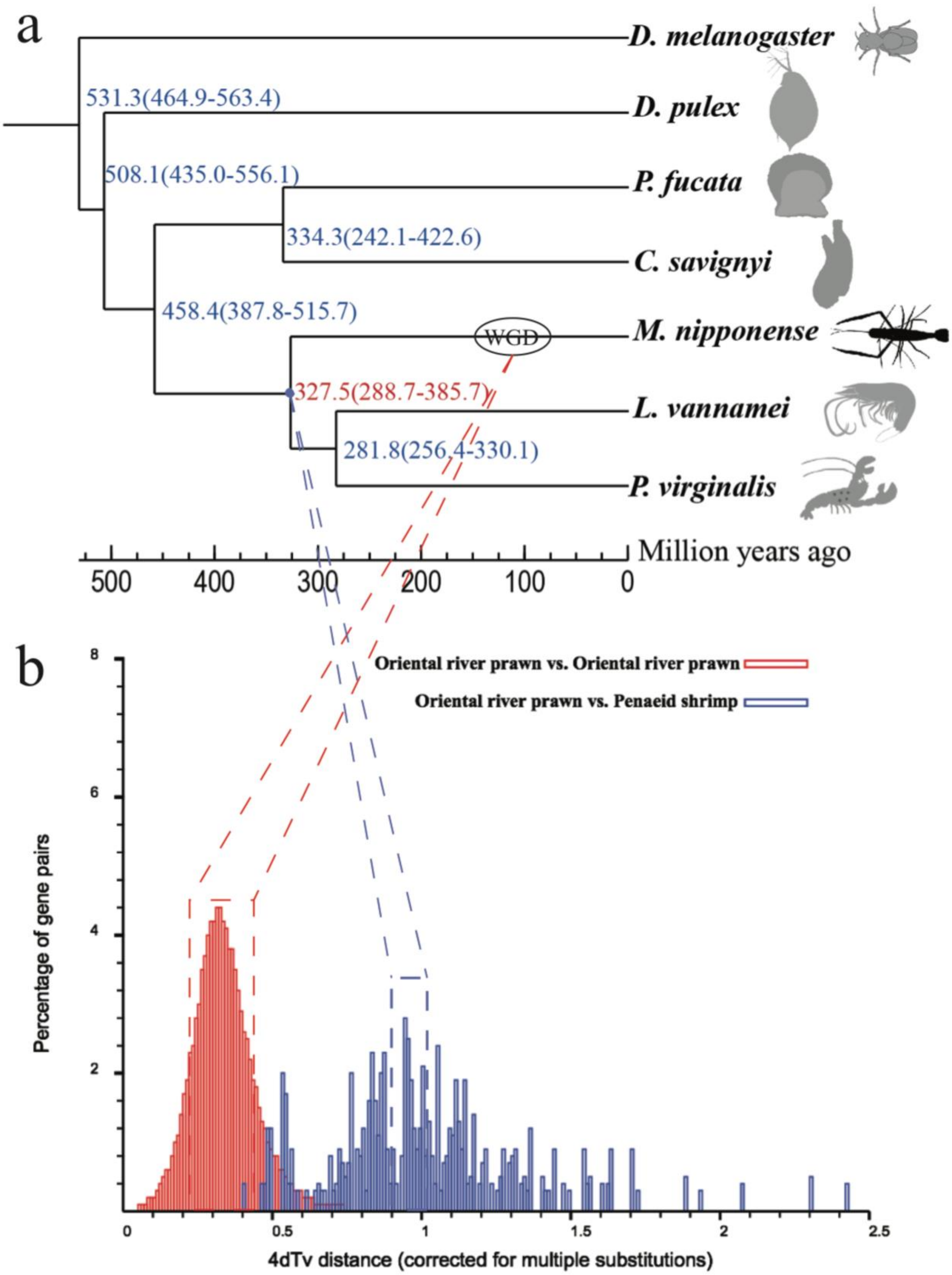

Figure 3. The divergence tree and the 4dTv results. (a) The divergence tree of seven representative species. The numbers with brackets represent confidence intervals. (b) The 4dTv distributions of oriental river prawn vs. oriental river prawn (red) and oriental river prawn vs. penaeid shrimp (blue).

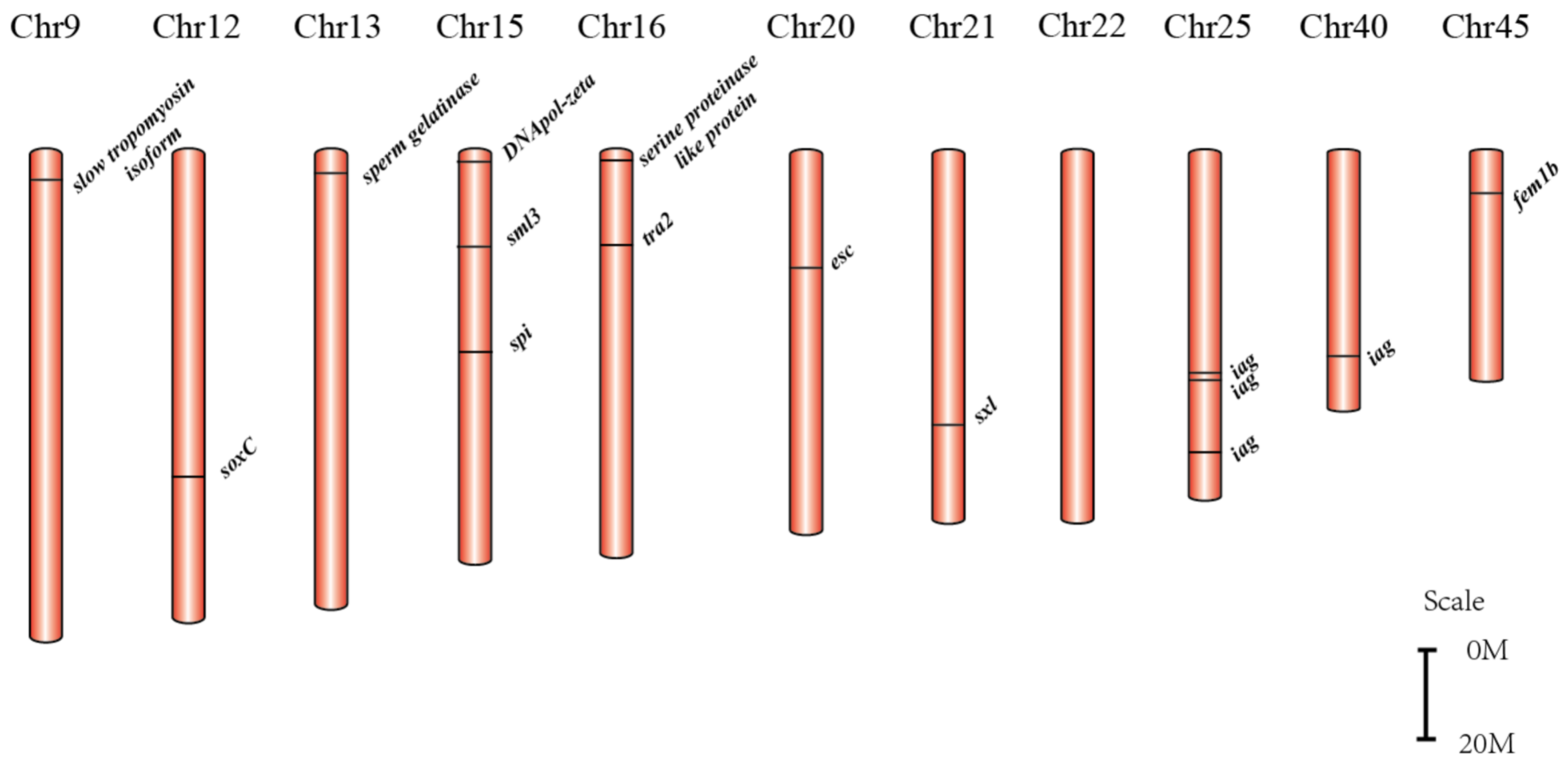

Figure 4. Sex-related candidate genes in the assembled oriental river prawn genome.

Reference

1. Ma K, Feng J, Lin J, Li J: **The complete mitochondrial genome of *Macrobrachium nipponense***. *Gene* 2011, **487**(2):160-165.
2. Yu HP, Miyake S: **Five species of the genus *Macrobrachium* (Crustacea, Decapoda, Palaemonidae) from Taiwan**. *Ohmu* 1972, **3**:45-55.
3. Jin Shubo ZY, Guan Haihong, Fu Hongtuo, Jiang Sufei, Xiong Yiwei, Qiao Hui, Zhang Wenyi, Gong Yongsheng, Wu Yan: **Histological observation of gonadal development during post-larva in oriental river prawn, *Macrobrachium nipponense***. *Chines Journal of Fisheries* 2016, **29**(04):11-16.
4. Li R, Yu C, Li Y, Lam T-W, Yiu S-M, Kristiansen K, Wang J: **SOAP2: an improved ultrafast tool for short read alignment**. *Bioinformatics* 2009, **25**(15):1966-1967.
5. Salmela L, Rivals E: **LoRDEC: accurate and efficient long read error correction**. *Bioinformatics* 2014, **30**(24):3506-3514.
6. Marçais G, Kingsford C: **A fast, lock-free approach for efficient parallel counting of occurrences of k-mers**. *Bioinformatics* 2011, **27**(6):764-770.
7. Shafin K, Pesout T, Lorig-Roach R, Haukness M, Olsen HE, Bosworth C, Armstrong J, Tigyi K, Maurer N, Koren S *et al*: **Nanopore sequencing and the Shasta toolkit enable efficient de novo assembly of eleven human genomes**. *Nature Biotechnology* 2020, **38**:1044-1053.
8. Zhu Y, Sun Z, Han Q, Liao L, Wang J, Bian C, Li J, Yan X, Liu Y, Shao C *et al*: **Human mesenchymal stem cells inhibit cancer cell proliferation by secreting DKK-1**. *Leukemia* 2009, **23**(5):925-933.
9. Walker BJ, Abeel T, Shea T, Priest M, Abouelliel A, Sakthikumar S, Cuomo CA, Zeng Q, Wortman J, Young SK *et al*: **Pilon: an integrated tool for comprehensive microbial variant detection and genome assembly improvement**. *PloS One* 2014, **9**(11):e112963.
10. Rao SS, Huntley MH, Durand NC, Stamenova EK, Bochkov ID, Robinson JT, Sanborn AL, Machol I, Omer AD, Lander ES *et al*: **A 3D map of the human genome at kilobase resolution reveals principles of chromatin looping**. *Cell* 2014, **159**(7):1665-1680.
11. Servant N, Varoquaux N, Lajoie BR, Viara E, Chen CJ, Vert JP, Heard E, Dekker J, Barillot E: **HiC-Pro: an optimized and flexible pipeline for Hi-C data processing**. *Genome Biology* 2015, **16**:259.

12. Durand NC, Shamim MS, Machol I, Rao SS, Huntley MH, Lander ES, Aiden EL: **Juicer provides a one-click system for analyzing loop-resolution Hi-C experiments**. *Cell Systems* 2016, **3**(1):95-98.

13. Dudchenko O, Batra SS, Omer AD, Nyquist SK, Hoeger M, Durand NC, Shamim MS, Machol I, Lander ES, Aiden AP *et al*: **De novo assembly of the *Aedes aegypti* genome using Hi-C yields chromosome-length scaffolds**. *Science* 2017, **356**(6333):92-95.

14. Durand NC, Robinson JT, Shamim MS, Machol I, Mesirov JP, Lander ES, Aiden EL: **Juicebox provides a visualization system for Hi-C contact maps with unlimited zoom**. *Cell Systems* 2016, **3**(1):99-101.

15. Abrusán G, Grundmann N, DeMester L, Makalowski W: **TEclass—a tool for automated classification of unknown eukaryotic transposable elements**. *Bioinformatics* 2009, **25**(10):1329-1330.

16. Xu Z, Wang H: **LTR_FINDER: an efficient tool for the prediction of full-length LTR retrotransposons**. *Nucleic Acids Research* 2007, **35**(suppl_2):W265-W268.

17. Tarailo-Graovac M, Chen N: **Using RepeatMasker to identify repetitive elements in genomic sequences**. *Current Protocols in Bioinformatics* 2009, **25**(1):4.10.11-14.10.14.

18. Jurka J, Kapitonov VV, Pavlicek A, Klonowski P, Kohany O, Walichiewicz J: **Repbase Update, a database of eukaryotic repetitive elements**. *Cytogenetic and Genome Research* 2005, **110**(1-4):462-467.

19. Benson G: **Tandem repeats finder: a program to analyze DNA sequences**. *Nucleic Acids Research* 1999, **27**(2):573-580.

20. Stanke M, Morgenstern B: **AUGUSTUS: a web server for gene prediction in eukaryotes that allows user-defined constraints**. *Nucleic Acids Research* 2005, **33**(suppl_2):W465-W467.

21. Mount DW: **Using the basic local alignment search tool (BLAST)**. *Cold Spring Harbor Protocols* 2007, **2007**(7):pdb. top17.

22. Birney E, Clamp M, Durbin R: **GeneWise and genomewise**. *Genome Research* 2004, **14**(5):988-995.

23. Kim D, Langmead B, Salzberg SL: **HISAT: a fast spliced aligner with low memory requirements**. *Nature Methods* 2015, **12**(4):357-360.

24. Trapnell C, Hendrickson DG, Sauvageau M, Goff L, Rinn JL, Pachter L: **Differential analysis of gene regulation at transcript resolution with RNA-seq**. *Nature Biotechnology* 2013, **31**(1):46-53.

25. Cantarel BL, Korf I, Robb SM, Parra G, Ross E, Moore B, Holt C, Alvarado AS, Yandell M: **MAKER: an easy-to-use annotation pipeline designed for emerging model organism genomes**. *Genome Research* 2008, **18**(1):188-196.
26. Boeckmann B, Bairoch A, Apweiler R, Blatter M-C, Estreicher A, Gasteiger E, Martin MJ, Michoud K, O'Donovan C, Phan I: **The SWISS-PROT protein knowledgebase and its supplement TrEMBL in 2003**. *Nucleic Acids Research* 2003, **31**(1):365-370.
27. Kulikova T, Aldebert P, Althorpe N, Baker W, Bates K, Browne P, van den Broek A, Cochrane G, Duggan K, Eberhardt R: **The EMBL nucleotide sequence database**. *Nucleic Acids Research* 2004, **32**(suppl_1):D27-D30.
28. Ogata H, Goto S, Sato K, Fujibuchi W, Bono H, Kanehisa M: **KEGG: Kyoto encyclopedia of genes and genomes**. *Nucleic Acids Research* 1999, **27**(1):29-34.
29. Zdobnov EM, Apweiler R: **InterProScan--an integration platform for the signature-recognition methods in InterPro**. *Bioinformatics* 2001, **17**(9):847-848.
30. Finn RD, Coggill P, Eberhardt RY, Eddy SR, Mistry J, Mitchell AL, Potter SC, Punta M, Qureshi M, Sangrador-Vegas A *et al*: **The Pfam protein families database: towards a more sustainable future**. *Nucleic Acids Research* 2016, **44**(D1):D279-285.
31. Attwood TK, Flower DR, Lewis AP, Mabey JE, Morgan SR, Scordis P, Selley JN, Wright W: **PRINTS prepares for the new millennium**. *Nucleic Acids Research* 1999, **27**(1):220-225.
32. Corpet F, Gouzy J, Kahn D: **Recent improvements of the ProDom database of protein domain families**. *Nucleic Acids Research* 1999, **27**(1):263-267.
33. Schultz J, Copley RR, Doerks T, Ponting CP, Bork P: **SMART: a web-based tool for the study of genetically mobile domains**. *Nucleic Acids Research* 2000, **28**(1):231-234.
34. Li L, Stoeckert CJ, Roos DS: **OrthoMCL: identification of ortholog groups for eukaryotic genomes**. *Genome Research* 2003, **13**(9):2178-2189.
35. Edgar RC: **MUSCLE: multiple sequence alignment with high accuracy and high throughput**. *Nucleic Acids Research* 2004, **32**(5):1792-1797.
36. Guindon S, Dufayard J-F, Lefort V, Anisimova M, Hordijk W, Gascuel O: **New algorithms and methods to estimate maximum-likelihood phylogenies: assessing the performance of PhyML 3.0**. *Systematic Biology* 2010, **59**(3):307-321.
37. Yang Z: **PAML: a program package for phylogenetic analysis by maximum likelihood**. *Computer applications in the biosciences* 1997, **13**(5):555-556.
38. Wang Y, Tang H, Debarry JD, Tan X, Li J, Wang X, Lee TH, Jin H, Marler B, Guo H

et al: **MCScanX: a toolkit for detection and evolutionary analysis of gene synteny and collinearity**. *Nucleic Acids Research* 2012, **40**(7):e49.

39. Robinson MD, McCarthy DJ, Smyth GK: **edgeR: a Bioconductor package for differential expression analysis of digital gene expression data**. *Bioinformatics* 2010, **26**(1):139-140.

40. Chen S, Yang P, Jiang F, Wei Y, Ma Z, Kang L: **De novo analysis of transcriptome dynamics in the migratory locust during the development of phase traits**. *PloS One* 2010, **5**(12):e15633.

41. Simao FA, Waterhouse RM, Ioannidis P, Kriventseva EV, Zdobnov EM: **BUSCO: assessing genome assembly and annotation completeness with single-copy orthologs**. *Bioinformatics* 2015, **31**(19):3210-3212.

42. Ma KY, Li JL, Qiu GF: **Identification of putative regulatory region of insulin-like androgenic gland hormone gene (IAG) in the prawn *Macrobrachium nipponense* and proteins that interact with IAG by using yeast two-hybrid system**. *General and Comparative Endocrinology* 2016, **229**:112-118.

43. Zhang YP, Qiao H, Zhang WY, Sun SM, Jiang SF, Gong YS, Xiong YW, Jin SB, Fu HT: **Molecular cloning and expression analysis of two sex-lethal homolog genes during development in the oriental river prawn, *Macrobrachium nipponense***. *Genetics and Molecular Research* 2013, **12**(4):4698-4711.

44. Wang Y, Jin S, Fu H, Qiao H, Sun S, Zhang W, Jiang S, Gong Y, Xiong Y, Wu Y: **Molecular cloning, expression pattern analysis, and in situ hybridization of a Transformer-2 gene in the oriental freshwater prawn, *Macrobrachium nipponense* (de Haan, 1849)**. *3 Biotech* 2019, **9**(6):205.

45. Ventura T, Manor R, Aflalo ED, Weil S, Raviv S, Glazer L, Sagi A: **Temporal silencing of an androgenic gland-specific insulin-like gene affecting phenotypical gender differences and spermatogenesis**. *Endocrinology* 2009, **150**(3):1278-1286.

46. Ventura T, Manor R, Aflalo ED, Weil S, Khalaila I, Rosen O, Sagi A: **Expression of an androgenic gland-specific insulin-like peptide during the course of prawn sexual and morphotypic differentiation**. *ISRN Endocrinology* 2011, **2011**:476283.

47. Rosen O, Manor R, Weil S, Gafni O, Linial A, Aflalo ED, Ventura T, Sagi A: **A sexual shift induced by silencing of a single insulin-like gene in crayfish: ovarian upregulation and testicular degeneration**. *PloS One* 2010, **5**(12):e15281.

48. Ventura T, Manor R, Aflalo E, Weil S, Rosen O, Sagi A: **Timing sexual differentiation: full functional sex reversal achieved through silencing of a single insulin-like gene**

in the prawn, *Macrobrachium rosenbergii*. *Biology of Reproduction* 2011, **86**:90.

49. Sagi A, Cohen D, Milner Y: **Effect of androgenic gland ablation on morphotypic differentiation and sexual characteristics of male freshwater prawns, *Macrobrachium rosenbergii***. *General and Comparative Endocrinology* 1990, **77**(1):15-22.

50. Wedekind C: **Demographic and genetic consequences of disturbed sex determination**. *Philosophical transactions of the Royal Society of London Series B, Biological Sciences* 2017, **372**(1729):20160326.

51. Gaofeng Q, Nanshan D, Wei L: **Chromosomal and karyological studies on the freshwater prawn (Crustacea, Decapoda)**. *Oceanologia Et Limnologia Sinica* 1994, **5**:493-498.

52. Justo CC, Murofushi M, Aida K, Hanyu I: **Karyological studies on the freshwater prawn *Macrobrachium rosenbergii***. *Aquaculture* 1991, **97**(4):327-334.

53. Choudhary N, Sharma R, Asthana S, Vyas P, Krishna G: **Development of karyotype and localization of cytogenetic markers in Dimua river prawn, *Macrobrachium villosimanus* (Tiwari, 1949)**. *Journal of Biological Sciences* 2013, **13**(6):507-513.

54. Mittal O, Dhall U: **Chromosome studies in three species of freshwater decapods (Crustacea)**. *Cytologia* 1971, **36**:633-638.

55. Phimphan S, Tanomtong A, Seangphan N, Sangpakdee W: **Chromosome studies on freshwater prawn, *Macrobrachium lanchesteri* (Decapoda, Palaemonidae) from Thailand**. *The Nucleus* 2019, **62**:77-82.

56. Molina WF, Costa GWWF, Cunha IMC, Bertollo LAC, Cioffi MB: **Molecular cytogenetic analysis in freshwater prawns of the genus *Macrobrachium* (Crustacea: Decapoda: Palaemonidae)**. *International Journal of Molecular Sciences* 2020, **21**(7):2595.

57. Yuan J, Gao Y, Zhang X, Wei J, Liu C, Li F, Xiang J: **Genome sequences of marine shrimp *Exopalaemon carinicauda* Holthuis provide insights into genome size evolution of Caridea**. *Marine Drugs* 2017, **15**:213.

58. Jin S, Hu Y, Fu H, Sun S, Jiang S, Xiong Y, Qiao H, Zhang W, Gong Y, Wu Y: **Analysis of testis metabolome and transcriptome from the oriental river prawn (*Macrobrachium nipponense*) in response to different temperatures and illumination times**. *Comparative Biochemistry and Physiology Part D, Genomics & proteomics* 2020, **34**:100662.

59. Jin S; Bian C; Jiang S; Han K; Xiong Y; Zhang W; Shi C; Qiao H; Gao Z; Li R; Huang

Y; Gong Y; You X; Fan G; Shi Q; Fu H. **Supporting data for "A chromosome-level genome assembly of the Oriental river prawn, *Macrobrachium nipponense*"**. *GigaScience* Database 2020. http://dx.doi.org/10.5524/100843
