# Supplementary material for: A chromosome-level genome assembly of the oriental river prawn, Macrobrachium nipponense
Source: Gigascience. 2021 Jan 18;10(1):giaa160. doi: 10.1093/gigascience/giaa160 (PMC7812440; doi:10.1093/gigascience/giaa160)
Supplement: giaa160_Response_to_Reviewer_Comments_Original_Submission [file giaa160_response_to_reviewer_comments_original_submission.pdf]

Author's Response To Reviewer Comments

Close

Reviewer #1:
The manuscript by Jin and colleagues reports the chromosome-scale assembly of the genome of the river prawn Macrobrachium nipponense, an economically important crustacean species, and investigates potential sex-related candidate genes which might serve as potential molecular markers for early sex determination.
In summary, the quality of the reported genome assembly was good, thanks to the combination of PacBio, Illumina PE and Omni-C libraries, and this will undoubtedly represent an important resource for the aquaculture sector. However, as a data note, I believe this work tried to include way more biological data than it should have, considering that such data (in particular the data concerning WGD) has not been appropriately analyzed and discussed.
While my detailed comments are appended below, I anticipate that I would strongly suggest the authors to reshape this MS as a data note, i.e. by purging the text from most of the parts linked with sex determination candidates (which remains, in my opinion, rather weak) and WGD (whose possible existence may be briefly mentioned).
Reply: Thanks for your instructive comment. Yes, we reshaped our manuscript in accordance with your advice. That is to say, we limited the descriptions of DEGs to a bare minimum (Line 301-311), and expanded the discussions of WGD and karyotype comparisons of Macrobrachium species in the Discussion section （lines 287-300）.

From a methodological point of view, it was quite difficult to ascertain whether all gDNA libraries were obtained from a single individual (as it should) or not. This is a key technical issue that needs to be solved first and foremost, as it may possibly affect the quality of the genome assembly itself.
Reply: You are right, gDNAs are usually extracted from a single individual for whole genome sequencing so as to minimize the adverse effects of polymorphisms. However, the pooled muscles from one specimen of our river prawn weighted up to 4 g, although the total body weight individually was at a range of 13.02 to 15.56 g. It is not sufficient to extract enough gDNAs from a single individual for the whole genome sequencing project. Thus, we had to pool the muscle tissues from 5 individuals for the practical works. These five prawns were born by the same parent pair and then cultivated by us in the same pond of our local aquaculture base. This is a popularly compromise way for small animals. Your reconsideration is appreciated.

The authors built a phylogenetic tree with a phylogenemic approach, which however lacks several methodological details and a few key crustacean species that should have been included. This type of analysis would most certainly not be sufficient to be included in a full paper, and it is not necessary in a data note article.
Reply: We quite agree with you to use more crustacean species for a better phylogenetic tree. We considered that a chromosome-level genome assembly with continuous scaffolds would be more appropriate for a comparative phylogenomic analysis. However, those key species you recommended are reported with low-quality assemblies. For example, Pandalus phatyceros (scaffold N50: 1,512 bp; NCBI accession number: GCA_005815305.1), Caridina multidentata (scaffold N50: 819 bp; GCA_002091895.1), and Palaemon carinicauda (scaffold N50: 962 bp; GCA_004011675.1/) are too fragmented to be used for this phylogenetic analysis.
By the way, more methodological details were provided in lines 195-197 for your reconsideration.

One of the key findings of this study (a WGD event that occurred in M. nipponense) has not been discussed at all, and its timing has not been investigated in sufficient detail, leaving the reader with more doubts than before. The authors did not report whether this was an expected finding and did not discuss this important data in relation with previously published literature, including karyotype studies carried out in multiple Macrobrachium species and cytogenetic estimates of c-value.
Reply: Thank you for your nice advice. Yes, it is done. Related discussions of WGD and karyotype studies were provided in the revised manuscript. Please see more details in lines 287-300 under the Discussion section.

I'm also not fully convinced by the DEG analysis, as the authors should have tried to put more emphasis on the reliability of the candidate genes identified, which would have been expected to be characterized by very high fold-change values. This type of information are unfortunately not available in the present version of the manuscript, which merely reports the enrichment of KEGG terms, with little attention to the fact that such terms are strongly biased towards model species. To discuss such data in a more reliable way, the authors should have considered the enrichment of GO terms and conserved domains as well. Again, discussing such aspects in a full paper would require much more attention, and the current presentation of these data is excessive for a data note article. I would suggest the authors to limit the reporting of DEGs to the bare minimum, stating that a few plausible sex-related gene candidates have been identified, but that these will require further independent validation.
Reply: Thanks for your instructive comments. According to your advice, we tried to limit the descriptions of DEGs to a bare minimum (Line 301-311), and stated more about a few sex-related candidate genes although these primary conclusions require in-depth validations. See more details in lines 272-284 and 307-311.

L48: approximate – approximately
Reply: Sorry for the mistake. Yes, it is done in line 47.

L63: some reads are here referred to the company name (e.g. PacBio), others to the platforms name (Hiseq), others to the library type (Hi-C). Please be more consistent: PacBio is fine (you may use Pacbio long reads), but replace the other two terms with "paired-end and Hi-C libraries processed on an Illumina platform".
Reply: Thanks for your nice advice. Yes, it is done. We revised this sentence as follows in lines 61-63.
In our present study, a chromosome-level genome assembly of the Oriental river prawn was constructed by integration of Pacbio long reads, Illumina short reads, and Hi-C sequencing data.

L69: why did the authors use multiple individuals to obtain a reference genome? In principle, it would be always preferable to extract gDNA from a single individual, in order to minimize the impact of polymorphisms in the assembly, especially when expected heterozygosity is high.
Reply: You are right, gDNAs are usually extracted from a single individual for whole genome sequencing so as to minimize the adverse effects of polymorphisms. However, the pooled muscles from one specimen of our river prawn weighted up to 4 g, although the total body weight individually was at a range of 13.02 to 15.56 g. It is not sufficient to extract enough gDNAs from a single individual for the whole genome sequencing project. Thus, we had to pool the muscle tissues from 5 individuals for the practical works. These five prawns were born by the same parent pair and then cultivated by us in the same pond of our local aquaculture base. This is a popularly compromise way for small animals. Your reconsideration is appreciated. See more details in lines 77-79.

L76: Some information is missing here, with the regards to the multiple individuals sampled. In particular, were the libraries generated through pulled samples from the five individuals, or where different individuals used to generate different libraries?
Reply: We pooled multiple individuals to generate different libraries. Thus, we revised this sentence as follows in lines 77-83.
Five individuals from each group were pooled, and muscle DNAs from pooled samples were extracted using a Nucleic Acid Kit (Qiagen, Germantown, MD, USA) in accordance with the manufacturer's instructions. The extracted gDNAs was then used for constructing libraries for Illumina (Illumina Inc., San Diego, CA, USA) and PacBio (Menlo Park, CA, USA) sequencing. According to the Illumina's instructions, seven paired-end libraries were constructed with the following insert sizes: 270 bp, 500 bp, 800 bp, 2 kb, 5 kb, 10 kb and 20 kb.

L108: this requires an important clarification. Sequencing data from two different libraries have been used here, but as I mentioned before it is unclear whether these two libraries have been obtained from pooled gDNA from multiple individuals or from single individuals. In the former case, a correct estimate of genome size (and heterozygosity) is not possible.
Reply: The pooled muscle tissues from individual prawn were up to 4 g, which is insufficient to extract enough gDNAs for the whole genome sequencing project. Thus, we pooled multiple individuals to generate different libraries. In fact, this compromise way has been applied frequently for many small animals, which provided accurate estimates of genome size in many previous reports (such as Liu K. et al., 2017, GigaScience, 6(4): giw012). See more details in lines 77-83.

L122: polish -> polishing
Reply: Sorry for the typo. It was corrected in line 130 of the revised manuscript.

L123: I would have expected to see some information concerning Hi-C library preparation and sequencing, which are not present here. This is an important point for a data note article.
Reply: Thanks for your instructive advice. Yes, it is done. More details regarding the Hi-C library preparation were provided in lines 110-113 and 131-142.

L152: "Ostreae Concha, Fucata martensii". There is an error here, as these are not correct scientific names. The authors probably refer to Pinctada fucata martnesii, and I don't know what Ostreae Concha is. If this is not a species, then the species count would be eight, not nine. Also penaeus should be Litopenaeus.
Reply: Sorry for the mistakes. We revised Ostreae Concha (oyster) as Crassostrea gigas in lines 164. Meanwhile, Pinctada fucata martensii was used to replace Fucata martensii, and Litopenaeus vannamei was also provided in lines 164-165 of the revised manuscript.

L164: "by aligning against". Please be more specific, as this is also a BLAST-based analysis.
Reply: Yes, we replaced it with "a BLAST-based analysis" in line 176.

L166: again, this is not an alignment, but rather a detection of HMM models.
Reply: Yes, it is done. See more details in lines 195-197.

L185: several details are missing here, including the molecular model of evolution set (and a description of how it has been selected).
Reply: Thanks for your nice advice. Yes, we added more details as follows in lines 195-197.
Alignments of these 'supergenes' were carried out to construct a phylogenetic tree by using the Maximum Likelihood method in PhyML (v3.0, RRID:SCR_014629) with the HKY85 model and default parameters [36].

L209: DEGs: which comparisons were investigated here? I guess between the two seasons, but please be more specific here.
Reply: Yes, you are right. We made modifications as follows in lines 220-223.
The Cuffdiff in the Cufflink package with parameters of "-FDR 0.05 – geometric-norm TRUE –c 10" was utilized to predict differentially expressed genes (DEGs) in the testis and androgenic gland between reproductive season and non-reproductive season.

L210: folds should be "fold change"
Reply: Yes, it is done in line 224 of the revised manuscript.

L221: missed -> missing
Reply: Yes, it is done (line 235).

L248: "four iag genes" -"four paralogous iag genes"?
Reply: Yes, it is done in line 262.

L249: rather than the number of genes, it would be much more interesting here to get to know the distance in MB between the 3 genes
Reply: Thanks for your good advice. Yes, the distance covering these three iag genes was calculated to be 17.34 Mb. This sentence was therefore revised as follows in line 263-264.
The distance covering the three iag genes was 17.34 Mb with prediction of 363 genes in this area.

L291: "lower organism"???
Reply: Sorry for the misleading description of "lower organism". We deleted this sentence in the revised manuscript.

Discussion: I found it very disappoint to find no mention at all about the predicted WGD event. This interesting finding is only mentioned in the results section, and not discussed at all. Was this expected? Are other WGD events known in Palaemonidae or Pleocyemata?
Reply: Sorry for this missing discussion for the WGD event. We added a paragraph of WGD in the discussion section (lines 295-300).

How do the number of assembled choromosomes and genome size compare with cytogenetic estimates? I see, from the animal genome size database, various c-value estimates from different Macrobrachium species, ranging from 6.48 to 22.16 (but data from M. nipponense is not available).

Reply: Thanks for your good question. In fact, there is no report of predicted genome size of M. nipponense from a flow cytometry experiment. We here estimated its genome size is about 4.6 Gb. However, from those genomic estimates in our previous genome works, the predicted genome sizes from a kmer-analysis are usually similar to the data from flow cytometry experiments. Therefore, we are confident about the estimate, although corresponding cytogenetic estimate is expected to be done whenever it is possible.

A number of studies exist concerning Macrobrachium karyotype. According to Damrongphol and colleagues (1991), the aploid number of chromosomes in M. rosenbergii is 59, and this value is confirmed in M. malcompsonii (Rebecca et al. 2020). M. lachesteri has n = 58 (Phimphan et al. 2018). Others, like M. acanthurus and M. amazonicum have n=49 (Molina et al. 2020), as reported here, but this should be mentioned in the manuscript.
However, I think a key point that would be worth mentioning is that the number of assembled chromosomes matches with the expected number from karyotype studies.
Reply: According to your advice, we cited the studies of Macrobrachium karyotype in lines 287-294.

More importantly, is there any clear evidence that Macrobrachium spp. has undergone a WGD event? I think this should be supported by much more substantiated evidence, which would allow a more precise timing for this event.
Reply: No. However, we provided few consistent evidences for the WGD event in our manuscript. At first, in Fig. 2b (pink lines), we found large numbers of synteny blocks that were evenly distributed in each chromosome. On the other hand, we predicted 4dtv values from the gene synteny blocks. We observed a remarkable peak for M. nipponense in Fig. 3b, which predicted that a recent WGD event had occurred in the M. nipponense genome. Subsequently, we reconstructed that the 4dtv peak (blue) for L. vannamei was located at 0.94 (in the X-axis), which is similar to previous reports; the 4dtv peak (red) of M. nipponense was at 0.335. Meanwhile, we predicted the divergence between M. nipponense and L. vannamei was about 327.5 million years ago. Therefore, the WGD event in M. nipponense may appear about 109.8 million years ago.

Honestly, the closest genomes included in this analysis were those of L. vannamei and P. virginalis, but these species are Pleocyemata and quite distantly related with M. nipponense. Why were not the genomes of Pandalus platyceros, Caridina multidentata and Palaemon carinicauda used, since these are evolutionarily closer and available?
Reply: Thanks for your good questions and instructive comments. We quite agree with you to use more crustacean species for a better phylogenetic tree. We considered that a chromosome-level genome assembly with continuous scaffolds would be more appropriate for a comparative phylogenomic analysis. However, those key species you recommended are reported with low-quality assemblies. For example, Pandalus phatyceros (scaffold N50: 1,512 bp; NCBI accession number: GCA_005815305.1), Caridina multidentata (scaffold N50: 819 bp; GCA_002091895.1), and Palaemon carinicauda (scaffold N50: 962 bp; GCA_004011675.1/) are too fragmented to be used for this phylogenetic analysis.

In summary, the WGD point should be revised and probably just mentioned as a possibility suggested by the analyses carried out, but whose timing needs to be properly investigated in future works.
Reply: Thanks for your advice. Yes, we added a brief discussion of the WGD in lines 295-300.

Table 2 is incomplete. What do these numbers represent? Fold change values?
Reply: You are right, the numbers in Table 2 represent fold changes. Related description was provided in Table 2.

Reviewer #2:
This genome note describes the chromosomal assembly of the Oriental river prawn. It appears to be well assembled into chromosomes with a high BUSCO score. There gene models are reasonable given the whole genome duplication but someone in the future may wish to redo the annotation with the more recent version of Maker (version3) as they use version 2 which does not take advantage of EVM. This is a significant advance in the genomic resources for this clade of organisms and I recommend that it be published with some minor revisions.
1) The use of the term "lower organism" should be avoided as it is an outdated term to imply there are move and less evolved organism in the tree of life when in fact all organisms are equally evolved. Please revise the following sentence.

"The Oriental river prawn is a lower organism; thus, it makes sense that sex-related genes
292 were not enriched in a special location (Figure 4)."
Reply: Thanks for your nice advice. This sentence was deleted in the revised manuscript.

2) There is a lot of significance place on this one region on chromosome 25 where 3 iag genes are located in which some of the gene models between these three genes are also differentially expressed between the sexes. Based on the evidence, I feel this language should be toned down in their discussion and conclusions. For example, this sentence.
"Thus, "Signal transduction and Endocrine system metabolic pathways", and the DEGs in these two metabolic pathways, might dramatically affect the process of male sex-differentiation and development in the Oriental river prawn."
Reply: Yes, we agree with you. According to the comments from Reviewer 1, we limited descriptions of DEGs to a bare minimum. This sentence was revised as follows in lines 307-311.
A few plausible sex-related candidate genes were identified, particularly after combining the analysis of genes on Chromosome 25 and differential transcription in testis and androgenic gland between the non-reproductive and reproductive seasons. However, these results require more independent validations.
Meanwhile, similar correction was realized in the Conclusion section (lines 319-322).

The paragraph above this sentence defines the functions of each gene and then proclaim a final sentence saying Thus it follows. Honestly, it is not terribly clear from the definitions of the DEG genes in the above paragraph how it justifies a Thus statement. Now that they have a genome they could resequenced or perform GBS on 50 males and 50 females and determine if there is a sex locus and use that as stronger evidence for linkage of these genes to the sex determining region and then explore genes in that region that may be involved in the observed sexual body size dimorphism. Now the authors have already performed significant work here and this would be above and beyond the current work. My point being that they have identified some genes of potential interest that could be followed up with additional experiments and that the language could be toned down a little to accent this point and not draw away the significant work contained within.
Reply: Thanks for your instructive advice. We have limited the descriptions of DEGs to a bare minimum, and toned down the conclusions in lines 307-311.
In this data note, we just provided some plausible sex-related gene candidates. In our further plan, we will determine the detailed functions of these genes through RACE cloning, qRT-PCR analysis, in situ hybridization, and CRISPER-Cas9 knock-out; meanwhile, we are planning to identify some sex-related loci in these genes.

3) I can't find JACEGS000000000 in genbank at NCBI. Please be sure to release this data.
Reply: Yes, the chromosome-level genome assembly is accessible now. Please try it once more for public availability.

Close
