# Supplementary material for: A chromosome-level genome assembly of the oriental river prawn, Macrobrachium nipponense
Source: Gigascience. 2021 Jan 18;10(1):giaa160. doi: 10.1093/gigascience/giaa160 (PMC7812440; doi:10.1093/gigascience/giaa160)
Supplement: giaa160_Response_to_Reviewer_Comments_Revision_1 [file giaa160_response_to_reviewer_comments_revision_1.pdf]

Author's Response To Reviewer Comments

Close

Reviewer #1: Thank you for providing a revised version of your MS along with detailed replies to my comments.
I find that most of my concerns have been adequately solved. However, a few additional modifications would be needed i[illegible]
I understand the rationale for using multiple individuals, and I appreciate the strategy of selecting multiple individuals orig[illegible]
minimize the issues linked with heterozygosity in the assembly. I have, however, a strong objection here, as k-mer-based [illegible]
reliable under such circumstances, due the possible non-perfect overlap of the peaks generated by gDNA from multiple in[illegible]
present in the pool from each individual are unlikely to be identical. I therefore strongly suggest the authors to remove th[illegible]
size estimates, as these would be only reliable with the analysis of Illumina reads generated from a single individual.
Reply: Thanks for your instructive comments. Sorry for the misleading descriptions in the previous version. In fact, the Il[illegible]
was generated from a single individual. We extracted gDNAs from the muscle of this individual, and constructed two short [illegible]
and 800 bp respectively for the Illumina sequencing. The whole genome size was then estimated to be 4.6 Gb by the k-m[illegible]
However, we thought the gDNAs from a single individual would be insufficient for whole genome sequencing. Therefore, w[illegible]
Illumina sequencing and another batch of five individuals for Pacbio Sequencing, in order to provide sufficient gDNAs for t[illegible]
sequencing. Please find more details in the revised manuscript (lines 80-93).

Line 197: how was the HKY85 model selected? Did the authors use ModelTest to identify the best-fitting model of molecul[illegible]
Reply: In our study, PhyML was employed to construct the phylogenetic tree with the default model HKY85. Thus, we trie[illegible]
revise the phylogenetic tree by using the jModelTest. The best-fitting model was identified as GTR. Based on the GTR mod[illegible]
(Drosophila_melanogaster,(Daphnia_pulex,((Pinctada_fucata,Ciona_savignyi),(Macrobrachium_nipponense,(Procambarus_[illegible]
However, this revised tree has the same topology as the previous one. After fossil calibration of divergence times with the[illegible]
observed that the revised tree has no difference from the previous one.

Figure 3a: please indicate what do the numbers indicated between brackets mean (confidence intervals, I guess)
Reply: Yes, you are right. The numbers within brackets represent confidence intervals. Thais sentence was added to the fi[illegible]

Lines 298-300: please improve the English language used here.
Reply: Thanks for your advice. Yes, it is done in lines 304-307 of the manuscript.

The WGD discussion remains unconvincing. The authors mentioned Yuan et al. 2017 (the E. carinicauda genome) stating [illegible]
characterized by a WGD event. Actually, both genome size (5.7Gb) and 2n chromosome number (90) do not indicate sign[illegible]
rosembergii. On the other hand, the number of genes is more than 2X higher in E. carinicauda. The quality of the E. carin[illegible]
lower than the genome reported in this study, so do the authors think that clues linked with WGD might have been misse[illegible]
also note that other Palaemonidae species have been previously shown to be characterized by very large genomes. For ex[illegible]
value > 10. Overall, I believe that the timing of this WGD event cannot be defined with certainty at the present time due [illegible]
information for many species, so I would appreciate if the authors could state more explicitly that many uncertainties abo[illegible]
Reply: Thanks for your comments. Yes, you are right; we really think the important clues of WGD event have been missin[illegible]
contig N50 of the E. carinicauda genome is only 263bp. These data are indeed fragmental to be used for gene identificatio[illegible]
synteny blocks in the E. carinicauda genome were missed.
For the genome size, rich repeat content could be a major reason for the genome expansion. Based on our experience, th[illegible]
more repeat content.
For the WGD analysis, we employed both 4dtv and synteny block analyses that were well-performed and classical method[illegible]
animal and plant genome reports. In the coming future, if there is any newly published genome assembly of a close shrim[illegible]
related WGD event/time.
According to your advice, we rewrote related sentences. Please find more details in lines 301-307.

Close
