# Supplementary material for: A chromosome-level genome assembly of the oriental river prawn, Macrobrachium nipponense
Source: Gigascience. 2021 Jan 18;10(1):giaa160. doi: 10.1093/gigascience/giaa160 (PMC7812440; doi:10.1093/gigascience/giaa160)
Supplement: giaa160_Reviewer_1_Report_Original_Submission — Marco Gerdol -- 10/7/2020 Reviewed [file giaa160_reviewer_1_report_original_submission.pdf]

Reviewer Report

Title: A chromosome-level genome assembly of the Oriental river prawn, Macrobrachium nipponense

Version: Original Submission **Date:** 10/7/2020

Reviewer name: Marco Gerdol

Reviewer Comments to Author:

The manuscript by Jin and colleagues reports the chromosome-scale assembly of the genome of the river prawn Macrobrachium nipponense, an evonomically important crustacean species, and investigates potential sex-related candidate genes which might serve as potential molecular markers for early sex determination.

In summary, the quality of the reported genome assembly was good, thanks to the combination of PacBio, Illumina PE and Omni-C libraries, and this will undoubtedly represent an important resource for the aquaculture sector. However, as a data note, I believe this work tried to include way more biological data than it should have, considering that such data (in particular the data concerning WGD) has not been appropriately analyzed and discussed.

While my detailed comments are appended below, I anticipate that I would strongly suggest the authors to reshape this MS as a data note, i.e. by purging the text from most of the parts linked with sex determination candidates (which remains, in my opinion, rather weak) and WGD (whose possible existence may be briefly mentioned).

From a methodological point of view, it was quite difficult to ascertain whether all gDNA libraries were obtained from a single individual (as it should) or not. This is a key technical issue that needs to be solved first and foremost, as it may possibly affect the quality of the genome assembly itself.

The authors built a phylogenetic tree with a phylogenemic approach, which however lacks several methodological details and a few key crustacean species that should have been included. This type of analysis would most certainly not be sufficient to be included in a full paper, and it is not necessary in a data note article.

One of the key findings of this study (a WGD event that occurred in M. nipponense) has not been discussed at all, and its timing has not been investigated in sufficient detail, leaving the reader with more doubts than before. The authors did not report whether this was an expected finding and did not discuss this important data in relation with previously published literature, including karyotype studies carried out in multiple Macrobrachium species and cytogenetic estimates of c-value.

I'm also not fully convinced by the DEG analysis, as the authors should have tried to put more emphasis on the reliability of the candidate genes identified, which would have been expected to be characterized by very high fold-change values. This type of information are unfortunately not available in the present version of the manuscript, which merely reports the enrichment of KEGG terms, with little attention to the fact that such terms are strongly biased towards model species. To discuss such data in a more reliable way, the authors should have considered the enrichment of GO terms and conserved domains as well. Again, discussing such aspects in a full paper would require much more attention, and the current presentation of these data is excessive for a data note article. I would suggest the authors to

limit the reporting of DEGs to the bare minimum, stating that a few plausible sex-related gene candidates have been identified, but that these will require further independent validation.
L48: approximate - approximately
L63: some reads are here referred to the company name (e.g. PacBio), others to the platforms name (Hiseq), others to the library type (Hi-C). Please be more consistent: PacBio is fine (you may use Pacbio long reads), but replace the other two terms with "paired-end and Hi-C libraries processed on an Illumina platform".
L69: why did the authors use multiple individuals to obtain a reference genome? In principle, it would be always preferable to extract gDNA from a single individual, in order to minimize the impact of polymorphisms in the assembly, especially when expected heterozygosity is high.
L76: Some information is missing here, with the regards to the multiple individuals sampled. In particular, were the libraries generated through pulled samples from the five individuals, or where different individuals used to generate different libraries?
L108: this requires an important clarification. Sequencing data from two different libraries have been used here, but as I mentioned before it is unclear whether these two libraries have been obtained from pooled gDNA from multiple individuals or from single individuals. In the former case, a correct estimate of genome size (and heterozygosity) is not possible.
L122: polish -> polishing
L123: I would have expected to see some information concerning Hi-C library preparation and sequencing, which are not present here. This is an important point for a data note article.
L152: "Ostreae Concha, Fucata martensii". There is an error here, as these are not correct scientific names. The authors probably refer to Pinctada fucata martnesii, and I don't know what Ostreae Concha is. If this is not a species, then the species count would be eight, not nine. Also penaeus should be Litopenaeus.
L164: "by aligning against". Please be more specific, as this is also a BLAST-based analysis.
L166: again, this is not an alignment, but rather a detection of HMM models
L185: several details are missing here, including the molecular model of evolution set (and a description of how it has been selected).
L209: DEGs: which comparisons were investigated here? I guess between the two seasons, but please be more specific here.
L210: folds should be "fold change"
L221: missed -> missing
L248: "four iag genes" -"four paralogous iag genes"?
L249: rather than the number of genes, it would be much more interesting here to get to know the distance in MB between the 3 genes.
L291: "lower organism"???
Discussion: I found it very disappoint to find no mention at all about the predicted WGD event. This interesting finding is only mentioned in the results section, and not discussed at all. Was this expected? Are other WGD events known in Palaemonidae or Pleocyemata? How do the number of assembled choromosomes and genome size compare with cytogenetic estimates?
I see, from the animal genome size database, various c-value estimates from different Macrobrachium species, ranging from 6.48 to 22.16 (but data from M. nipponense is not available).

A number of studies exist concerning Macrobrachium karyotype. According to Damrongphol and colleagues (1991), the aploid number of chromosomes in M. rosenbergii is 59, and this value is confirmed in M. malcompsonii (Rebecca et al. 2020). M. lachesteri has n = 58 (Phimphan et al. 2018). Others, like M. acanthurus and M. amazonicum have n=49 (Molina et al. 2020), as reported here, but this should be mentioned in the manuscript.
More importantly, is there any clear evidence that Macrobrachium spp. has undergone a WGD event? I think this should be supported by much more substantiated evidence, which would allow a more precise timing for this event.
Honestly, the closest genomes included in this analysis were those of L. vannamei and P. virginalis, but these species are Pleocyemata and quite distantly related with M. nipponense. Why were not the genomes of Pandalus platyceros, Caridina multidentata and Palaemon carinicauda used, since these are evolutionarily closer and available?
In summary, the WGD point should be revised and probably just mentioned as a possibility suggested by the analyses carried out, but whose timing needs to be properly investigated in future works.
However, I think a key point that would be worth mentioning is that the number of assembled chromosomes matches with the expected number from karyotype studies.
Table 2 is incomplete. What do these numbers represent? Fold change values?

Level of Interest

Please indicate how interesting you found the manuscript: Choose an item.

Quality of Written English

Please indicate the quality of language in the manuscript: Choose an item.

Declaration of Competing Interests

Please complete a declaration of competing interests, considering the following questions:

- Have you in the past five years received reimbursements, fees, funding, or salary from an organisation that may in any way gain or lose financially from the publication of this manuscript, either now or in the future?
- Do you hold any stocks or shares in an organisation that may in any way gain or lose financially from the publication of this manuscript, either now or in the future?
- Do you hold or are you currently applying for any patents relating to the content of the manuscript?
- Have you received reimbursements, fees, funding, or salary from an organization that holds or has applied for patents relating to the content of the manuscript?
- Do you have any other financial competing interests?
- Do you have any non-financial competing interests in relation to this paper?

If you can answer no to all of the above, write 'I declare that I have no competing interests' below. If your reply is yes to any, please give details below.

'I declare that I have no competing interests

I agree to the open peer review policy of the journal. I understand that my name will be included on my report to the authors and, if the manuscript is accepted for publication, my named report including any attachments I upload will be posted on the website along with the authors' responses. I agree for my report to be made available under an Open Access Creative Commons CC-BY license (http://creativecommons.org/licenses/by/4.0/). I understand that any comments which I do not wish to be included in my named report can be included as confidential comments to the editors, which will not be published.

Choose an item.

To further support our reviewers, we have joined with Publons, where you can gain additional credit to further highlight your hard work (see: https://publons.com/journal/530/gigascience). On publication of this paper, your review will be automatically added to Publons, you can then choose whether or not to claim your Publons credit. I understand this statement.

Yes Choose an item.
