# Supplementary material for: A chromosome-level genome assembly of the oriental river prawn, Macrobrachium nipponense
Source: Gigascience. 2021 Jan 18;10(1):giaa160. doi: 10.1093/gigascience/giaa160 (PMC7812440; doi:10.1093/gigascience/giaa160)
Supplement: giaa160_Reviewer_1_Report_Revision_1 — Marco Gerdol -- 11/15/2020 Reviewed [file giaa160_reviewer_1_report_revision_1.pdf]

Reviewer Report

Title: A chromosome-level genome assembly of the Oriental river prawn, Macrobrachium nipponense

Version: Revision 1 **Date:** 11/15/2020

Reviewer name: Marco Gerdol

Reviewer Comments to Author:

Thank you for providing a revised version of your MS along with detailed replies to my comments.
I find that most of my concerns have been adequately solved. However, a few additional modifications would be needed in my opinion.
I understand the rationale for using multiple individuals, and I appreciate the strategy of selecting multiple individuals originated from the same parents to minimize the issues linked with heterozygosity in the assembly. I have, however, a strong objection here, as k-mer-based estimates of genome size are not reliable under such circumstances, due the possible non-perfect overlap of the peaks generated by gDNA from multiple individuals, as the quantity of gDNA present in the pool from each individual are unlikely to be identical. I therefore strongly suggest the authors to remove the mention of k-mer based genome size estimates, as these would be only reliable with the analysis of Illumina reads generated from a single individual.
Line 197: how was the HKY85 model selected? Did the authors use ModelTest to identify the best-fitting model of molecular evolution?
Figure 3a: please indicate what do the numbers indicated between brackets mean (confidence intervals, I guess)
Lines 298-300: please improve the English language used here.
The WGD discussion remains unconvincing. The authors mentioned Yuan et al. 2017 (the E. carinicauda genome) stating that this genome was not characterized by a WGD event. Actually, both genome size (5.7Gb) and 2n chromosome number (90) do not indicate significant differences compared with M. rosembergii. On the other hand, the number of genes is more than 2X higher in E. carinicauda. The quality of the E. carinicauda genome assembly was much lower than the genome reported in this study, so do the authors think that clues linked with WGD might have been missed in the study by Yuan et al.? Please also note that other Palaemonidae species have been previously shown to be characterized by very large genomes. For example Palemon serratus, with a c-value > 10. Overall, I believe that the timing of this WGD event cannot be defined with certainty at the present time due to the lack of genome and karyotype information for many species, so I would appreciate if the authors could state more explicitly that many uncertainties about this inference remain.

Level of Interest

Please indicate how interesting you found the manuscript: Choose an item.

Quality of Written English

Please indicate the quality of language in the manuscript: Choose an item.

Declaration of Competing Interests

Please complete a declaration of competing interests, considering the following questions:

- Have you in the past five years received reimbursements, fees, funding, or salary from an organisation that may in any way gain or lose financially from the publication of this manuscript, either now or in the future?
- Do you hold any stocks or shares in an organisation that may in any way gain or lose financially from the publication of this manuscript, either now or in the future?
- Do you hold or are you currently applying for any patents relating to the content of the manuscript?
- Have you received reimbursements, fees, funding, or salary from an organization that holds or has applied for patents relating to the content of the manuscript?
- Do you have any other financial competing interests?
- Do you have any non-financial competing interests in relation to this paper?

If you can answer no to all of the above, write 'I declare that I have no competing interests' below. If your reply is yes to any, please give details below.

I declare that I have no competing interests

I agree to the open peer review policy of the journal. I understand that my name will be included on my report to the authors and, if the manuscript is accepted for publication, my named report including any attachments I upload will be posted on the website along with the authors' responses. I agree for my report to be made available under an Open Access Creative Commons CC-BY license (http://creativecommons.org/licenses/by/4.0/). I understand that any comments which I do not wish to be included in my named report can be included as confidential comments to the editors, which will not be published.

Choose an item.

To further support our reviewers, we have joined with Publons, where you can gain additional credit to further highlight your hard work (see: https://publons.com/journal/530/gigascience). On publication of this paper, your review will be automatically added to Publons, you can then choose whether or not to claim your Publons credit. I understand this statement.

Yes Choose an item.
