# Supplementary material for: A chromosome-level genome assembly of the oriental river prawn, Macrobrachium nipponense
Source: Gigascience. 2021 Jan 18;10(1):giaa160. doi: 10.1093/gigascience/giaa160 (PMC7812440; doi:10.1093/gigascience/giaa160)
Supplement: giaa160_Reviewer_2_Report_Original_Submission — Andrew Severin -- 10/6/2020 Reviewed [file giaa160_reviewer_2_report_original_submission.pdf]

Reviewer Report

Title: A chromosome-level genome assembly of the Oriental river prawn, Macrobrachium nipponense

Version: Original Submission **Date:** 10/6/2020

Reviewer name: Andrew Severin

Reviewer Comments to Author:

This genome note describes the chromosomal assembly of the Oriental river prawn. It appears to be well assembled into chromosomes with a high BUSCO score. There gene models are reasonable given the whole genome duplication but someone in the future may wish to redo the annotation with the more recent version of Maker (version3) as they use version 2 which does not take advantage of EVM. This is a significant advance in the genomic resources for this clade of organisms and I recommend that it be published with some minor revisions.

1) The use of the term "lower organism" should be avoided as it is an outdated term to imply there are move and less evolved organism in the tree of life when in fact all organisms are equally evolved. Please revise the following sentence.
"The Oriental river prawn is a lower organism; thus, it makes sense that sex-related genes
292 were not enriched in a special location (Figure 4)."

2) There is a lot of significance place on this one region on chromosome 25 where 3 iag genes are located in which some of the gene models between these three genes are also differentially expressed between the sexes. Based on the evidence, I feel this language should be toned down in their discussion and conclusions. For example, this sentence.
"Thus,
318 "Signal transduction and Endocrine system metabolic pathways", and the DEGs in these two
319 metabolic pathways, might dramatically affect the process of male sex-differentiation and
320 development in the Oriental river prawn."
The paragraph above this sentence defines the functions of each gene and then proclaim a final sentence saying Thus it follows. Honestly, it is not terribly clear from the definitions of the DEG genes in the above paragraph how it justifies a Thus statement. Now that they have a genome they could resequenced or perform GBS on 50 males and 50 females and determine if there is a sex locus and use that as stronger evidence for linkage of these genes to the sex determining region and then explore genes in that region that may be involved in the observed sexual body size dimorphism. Now the authors have already performed significant work here and this would be above and beyond the current work. My point being that they have identified some genes of potential interest that could be followed up with additional experiments and that the language could be toned down a little to accent this point and not draw away the significant work contained within.

3) I can't find JACEGS000000000 in genbank at NCBI. Please be sure to release this data.

Level of Interest

Please indicate how interesting you found the manuscript: Choose an item.

Quality of Written English

Please indicate the quality of language in the manuscript: Choose an item.

Declaration of Competing Interests

Please complete a declaration of competing interests, considering the following questions:

- Have you in the past five years received reimbursements, fees, funding, or salary from an organisation that may in any way gain or lose financially from the publication of this manuscript, either now or in the future?
- Do you hold any stocks or shares in an organisation that may in any way gain or lose financially from the publication of this manuscript, either now or in the future?
- Do you hold or are you currently applying for any patents relating to the content of the manuscript?
- Have you received reimbursements, fees, funding, or salary from an organization that holds or has applied for patents relating to the content of the manuscript?
- Do you have any other financial competing interests?
- Do you have any non-financial competing interests in relation to this paper?

If you can answer no to all of the above, write 'I declare that I have no competing interests' below. If your reply is yes to any, please give details below.

I declare that I have no competing interests

I agree to the open peer review policy of the journal. I understand that my name will be included on my report to the authors and, if the manuscript is accepted for publication, my named report including any attachments I upload will be posted on the website along with the authors' responses. I agree for my report to be made available under an Open Access Creative Commons CC-BY license (http://creativecommons.org/licenses/by/4.0/). I understand that any comments which I do not wish to be included in my named report can be included as confidential comments to the editors, which will not be published.

Choose an item.

To further support our reviewers, we have joined with Publons, where you can gain additional credit to further highlight your hard work (see: https://publons.com/journal/530/gigascience). On publication of this paper, your review will be automatically added to Publons, you can then choose whether or not to claim your Publons credit. I understand this statement.

Yes Choose an item.
